# Evaluation of Combination Strategies for the A_2A_R Inhibitor AZD4635 Across Tumor Microenvironment Conditions *via* a Systems Pharmacology Model

**DOI:** 10.3389/fimmu.2021.617316

**Published:** 2021-03-02

**Authors:** Veronika Voronova, Kirill Peskov, Yuri Kosinsky, Gabriel Helmlinger, Lulu Chu, Alexandra Borodovsky, Richard Woessner, Kris Sachsenmeier, Wenlin Shao, Rakesh Kumar, Gayle Pouliot, Melinda Merchant, Holly Kimko, Ganesh Mugundu

**Affiliations:** ^1^ M&S Decisions LLC, Moscow, Russia; ^2^ Computational Oncology Group, I.M. Sechenov First Moscow State Medical University, Moscow, Russia; ^3^ Clinical Pharmacology and Quantitative Pharmacology, BioPharmaceuticals R&D, AstraZeneca R&D Boston, Boston, MA, United States; ^4^ Translational Medicine, AstraZeneca R&D Boston, Waltham, MA, United States; ^5^ Oncology, BioPharmaceuticals R&D, AstraZeneca R&D Boston, Boston, MA, United States

**Keywords:** adenosine, PD-L1, checkpoint inhibitors, immunotherapy, mathematical modeling, combination strategies, treatment optimization

## Abstract

**Background:**

Adenosine receptor type 2 (A_2A_R) inhibitor, AZD4635, has been shown to reduce immunosuppressive adenosine effects within the tumor microenvironment (TME) and to enhance the efficacy of checkpoint inhibitors across various syngeneic models. This study aims at investigating anti-tumor activity of AZD4635 alone and in combination with an anti-PD-L1-specific antibody (anti-PD-L1 mAb) across various TME conditions and at identifying, *via* mathematical quantitative modeling, a therapeutic combination strategy to further improve treatment efficacy.

**Methods:**

The model is represented by a set of ordinary differential equations capturing: 1) antigen-dependent T cell migration into the tumor, with subsequent proliferation and differentiation into effector T cells (Teff), leading to tumor cell lysis; 2) downregulation of processes mediated by A_2A_R or PD-L1, as well as other immunosuppressive mechanisms; 3) A_2A_R and PD-L1 inhibition by, respectively, AZD4635 and anti-PD-L1 mAb. Tumor size dynamics data from CT26, MC38, and MCA205 syngeneic mice treated with vehicle, anti-PD-L1 mAb, AZD4635, or their combination were used to inform model parameters. Between-animal and between-study variabilities (BAV, BSV) in treatment efficacy were quantified using a non-linear mixed-effects methodology.

**Results:**

The model reproduced individual and cohort trends in tumor size dynamics for all considered treatment regimens and experiments. BSV and BAV were explained by variability in T cell-to-immunosuppressive cell (ISC) ratio; BSV was additionally driven by differences in intratumoral adenosine content across the syngeneic models. Model sensitivity analysis and model-based preclinical study simulations revealed therapeutic options enabling a potential increase in AZD4635-driven efficacy; *e.g.*, adoptive cell transfer or treatments affecting adenosine-independent immunosuppressive pathways.

**Conclusions:**

The proposed integrative modeling framework quantitatively characterized the mechanistic activity of AZD4635 and its potential added efficacy in therapy combinations, across various immune conditions prevailing in the TME. Such a model may enable further investigations, *via* simulations, of mechanisms of tumor resistance to treatment and of AZD4635 combination optimization strategies.

## Introduction

Adenosine is a purine nucleoside which, under ischemic conditions, may accumulate in the extracellular microenvironment and be involved in the downregulation of inflammation processes (1). While this downregulation may be desired in some pathological states (*e.g.*, myocardial infarction), it also reduces anti-tumor immune responses and may potentially limit the efficacy of immuno-oncology (IO) therapeutic agents. In the tumor microenvironment (TME), hypoxia is followed by cancer cell necrosis, with a subsequent release of adenosine and its precursors into the extracellular space. Hypoxia-induced activation of ectonucleases CD39 and CD73—enzymes producing adenosine—may result in a ~10-fold increase of extracellular adenosine within tumors, as compared to normal tissue (2–4). Amounting levels of adenosine activate A_2A_R and A_2B_R, receptors expressed on the surface of various immune cell populations. Adenosine has been shown to impair antigen-presenting cell proliferation, decrease effector T cells (Teff) activation, induce Treg activation, skew macrophage polarization from a pro-inflammatory to an anti-inflammatory and angiogenic phenotype, and inhibit NK cell activity (5, 6).

Various therapeutic approaches aiming at blocking adenosine effects have been extensively tested preclinically (7). These include blocking of either adenosine production with CD73 or CD39 monoclonal antibodies (mAb) (8–11) or downstream adenosine effects with A_2A_R or A_2B_R inhibitors (12–15). These interventions demonstrated efficacy as monotherapy or combination with other IO agents [PD-(L)1 mAb, CTLA-4 mAb, and dendritic cell (DC) vaccines] and/or chemotherapy (5, 6, 8–10, 12–14) and are now being tested in clinical trials (16). AZD4635 is a potent and selective A_2A_R inhibitor shown to enhance anti-tumor immunity *via* activation of antigen presentation and restoration of Teff functions (17). Treatment with AZD4635 alone and in combination with an anti-PD-L1 mAb was associated with reduction of tumor burden in phase I clinical trials in subjects with refractory solid tumors (18) and is currently entering a phase II study in subjects with metastatic castration-resistant prostate cancer [https://clinicaltrials.gov/ct2/show/NCT04089553].

While initial clinical trials demonstrated therapeutic potential of A_2A_R blockade (19), ongoing research is aimed at further improvement of treatment outcomes. The latter can be achieved by evaluation of various combination options in the preclinical setting and the identification of patients who are likely to respond to treatment in early trials. The use of RNA-based, gene signature-derived multivariate scores, associated with treatment effects, may be a useful tool for such patient selection; two adenosine signatures have been recently identified as potentially good prognostic and predictive markers (19, 20).

The integrative modeling framework described here, also referred to as quantitative systems pharmacology (QSP) modeling, allows for the quantitative characterization of a compound’s mechanism of action, with a time-dependent description of cell interactions within the TME upon compound dosing, all linked to endpoint(s) of interest (namely, tumor size dynamics). Simulations may then be used to mechanistically explore features of resistance to treatment across various sets of cellular and molecular immune conditions prevailing in the TME, based on the integration of extensive preclinical datasets into the modeling framework (21). The core structure of the QSP model captures key time-dependent steps of the cancer immunity cycle, including antigen-presentation, migration and expansion of tumor-specific lymphocyte clones, and immunogenic tumor cell death (22). Each step in this cycle may be modulated by various immunosuppressive forces, depending on the TME type, and the effects of various therapeutic IO agents on these forces quantified. The QSP model may thus be used to characterize (over time, in terms of relative levels, and for various sets of prevailing TME conditions) fundamental mechanisms of anti-tumor immune responses for dosing schemes of choice, in mono- and combination therapy settings. Another distinctive feature of the proposed framework is the application of a non-linear mixed effect modeling (NLME) methodology (23), enabling evaluation of between-animal and between-study variabilities (BAV, BSV) in the observed tumor size dynamics. Such a model includes a statistical component, capturing variations in immune system parameters across the animals, driving differences in treatment efficacy, which is essential in the analysis of immunotherapeutic interventions, where a wide range of responses are often observed. The present research aimed at developing, specifically, a QSP NLME model based upon four independent preclinical studies evaluating the efficacy of an anti-PD-L1 mAb, AZD4635, or their combination, in three syngeneic murine models characterized by different prevailing TME conditions (CT26, MC38 and MCA205). Upon model qualification, predictive simulations were then performed to identify determinants of AZD4635 activity and to prioritize treatment strategies, given specific sets of TME conditions.

## Materials and Methods

### Experimental Data

Multiple sets of experimental data were used for model parameter estimation and model qualification:1) A_2A_R binding affinities for AZD4635 and adenosine, obtained from *in vitro* functional cAMP studies (17); 2) plasma AZD4635 profiles available from 9 Balb/c mice treated with single oral doses of 10, 25, or 50 mg/kg; 3) total adenosine measurements and tumor size dynamics data measured in CT26, MC38, and MCA205 syngeneic mouse models (**Supplementary Materials, Figure S1**). The final dataset was comprised of pooled data from four experimental studies, with individual animal tumor measurements from 116 Balb/c mice (17). Three studies were performed in 116 animals injected subcutaneously with 1x10^6^ CT26, 1x10^6^ MC38, or 5x10^5^ MCA205 tumor cells. Treatment was initiated when tumor size reached ~50-90 mm^2^ and continued for 2 weeks. The following treatment regimens were tested: 1) control isotype mAb; 2) an anti-PD-L1 mAb at 5 mg/kg twice–weekly (BIW); 3) AZD4635 50 mg/kg twice-daily (BID); 4) combination of the aforementioned anti-PD-L1 mAb and AZD4635 treatment regimens. In an additional dose-finding study, 40 mice inoculated with 5x10^5^ MCA205 tumor cells were treated with: 1) a control isotype Ab; 2) an anti-PD-L1 mAb at 5 mg/kg BIW, alone or in combination with: 3) 10 mg/kg, 4) 25 and 50 mg/kg twice daily AZD4635 (17).

### Mathematical Modeling

**Figure 1:**
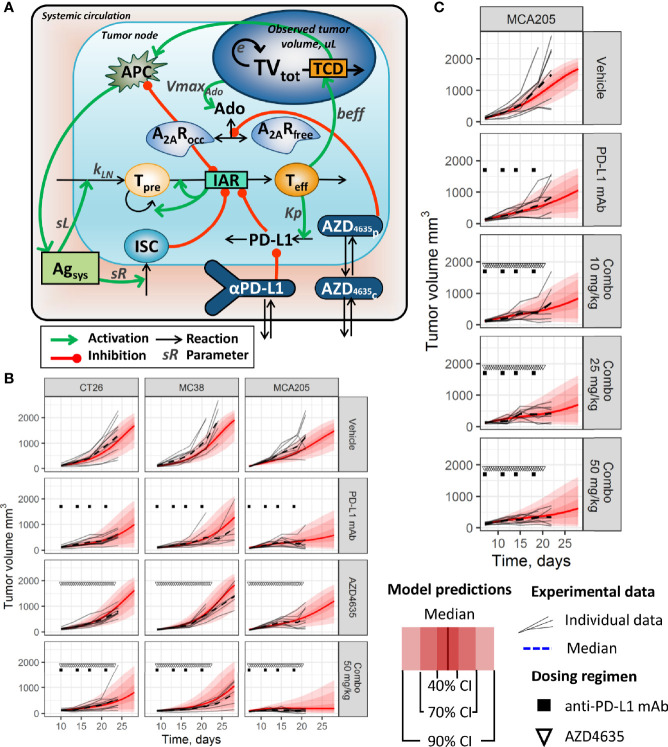
**(A)** Simplified model schematic. Abbreviations used: Ado, adenosine level; A_2A_R, unbound A_2A_R level; A_2A_Rocc, occupied A_2A_R level; Agsys, systemic level of tumor antigen; AZD4635c, AZD4635 concentration in central compartment; AZD4635p, AZD4635 concentration in peripheral compartment; IAR, immune activation rate; ISC, component of immunosuppressive cells in TME; PD-L1, PD-L1 immunosuppressive component in TME; αPD-L1, an anti-PD-L1 mAb level in central compartment; nTeff, non-differentiated T-cells; dTeff, cytotoxic effector T-cells; TCD, tumor cell death; TVtot, total tumor volume. **(B, C)** Distributions of population model predictions and corresponding tumor size dynamics data from **(B)** efficacy studies in CT26, MC38, and MCA205 syngeneic models (dosing regimen: 5 mg/kg anti-PD-L1 mAb BW; 50 mg/kg AZD4635 BID). **(C)** A dose-finding study in MCA205 syngeneic model (dosing regimen: 5 mg/kg anti-PD-L1 mAb BW; 10–50 mg/kg AZD4635 BID).

The core of the QSP model, which characterizes key stages of the anti-tumor immune response, was taken from our group’s previous work (21). The model structure consisted of ordinary differential equations (ODE) and functions describing the interplay among key elements of the cancer immunity cycle (**Figure 1A**); these were chosen based on experimental measurements or on existing mathematical models. Briefly, an immune response was triggered by tumor antigens released during immunogenic tumor cell death (TCD). This process was followed by antigen presentation, migration of tumor-specific lymphocyte precursors (nTeff), and their subsequent activation, proliferation and differentiation into professional cytotoxic effector T-lymphocytes (dTeff) capable of performing tumor cell kill - thereby intensifying tumor antigen supply. The immunosuppressive TME was represented by various immunosuppressive cells (ISC), which would infiltrate tumor tissue in response to antigen presentation, downregulate effector T cells, as well as activate negative Teff-mediated PD-(L)1 feedback. The latter can be blocked by an anti-PD-L1 mAb; the dynamics of PD-L1 inhibition was determined by the plasma mAb profile, captured by a corresponding pharmacokinetic (PK) sub-model.

The model structure also included a description of adenosine-mediated immunosuppression based on experimental information collected from the literature and summarized in the **Supplementary Materials, Section 1**. The model considered adenosine release from dying cancer cells and its binding to A_2A_R within the TME, followed by downregulation of antigen presentation as well as a decrease in T lymphocyte activation. Immunosuppressive adenosine effects were diminished, in the model, by administration of AZD4635 *via* competition with adenosine for the A_2A_R binding site. A two-compartment PK sub-model was used to reproduce the AZD4635 plasma profile. A detailed description of all model equations is provided in the **Supplementary Materials, Section 2**.

An NLME approach was used to link variability in observed tumor dynamics with differences in cellular or molecular functional aspects of the immune system. The following set of parameters describing variability were introduced into the model: 1) fixed constants (similar across all animals); 2) random effect coefficients, capturing BAV; 3) covariate coefficients, reflecting BSV; 4) residual error model parameters, characterizing unexplained variability across experimental measurements. A total of 43 parameters were used (**Table S2**); 11 of these were taken from a QSP IO model developed previously by Kosinsky et al. (21). An additional 10 parameters were fixed based on further experimental data. A total of 6 AZD4635 pharmacokinetic parameters were estimated using plasma PK profiles, and two parameters reflecting binding affinities of AZD4635 and adenosine to A_2A_R were estimated using *in vitro* data. Values for the remaining 18 parameters were estimated using individual tumor size dynamics data described above (*Experimental Data* section). Covariate coefficients, which characterized intratumoral adenosine accumulation (Vmax_Ado_) and initial tumor volume (TVtot_0_) were set based on, respectively, adenosine measurements obtained from syngeneic models and numbers of inoculated tumor cells (17). Selection of additional covariates as well as random effects was performed using a covariate search routine (forward-addition method). Based on the quality of experimental data reproduction—which was evaluated using an objective function value (OFV), most of the BSV and BAV could be explained by variability in parameters sL and sR, which captured influx of, respectively, immunoactive and immunosuppressive cells into the TME (**Supplementary Table S1**).

The quality of the developed model was evaluated using multiple criteria (24, 25). These include: i) analyses of diagnostic plots (population and individual predictions *vs.* experimental observations, distribution of population, and individual weighted residuals, *etc.*—see (25) and **Supplementary Figures S5** and **S6**; ii) precision and identifiability of parameter estimates based on estimated relative standard error values—see (26) and **Supplementary Table S2**; and iii) minimization of random effects and residual errors. Automatic parameter estimation and model analyses were based on a stochastic approximation expectation maximization (SAEM) algorithm and performed using the Monolix^®^ 2019R1 software (Lixoft, Antony, France). Model simulation runs and visualizations were performed in the R software version 3.4.1, using packages mlxR and ggplot2. The model code and the R-based scripts, used for the simulations, are available in the GitHub repository [https://github.com/VeronikaVor2/IO-QSP-model-adenosine-].

## Results

### Data Reproduction by the Proposed Model

The model provided adequate and unbiased reproduction of tumor dynamics distributions for the various treatment conditions and across preclinical experiments (**Figures 1B, C**; corresponding model diagnostic plots are available in the **Supplementary Materials, Section 4** and **Figures S2–S4**). As observed based on both experimental data and model predictions, the anti-PD-L1 mAb demonstrated limited efficacy in the evaluated syngeneic models, while there was an increase in efficacy when combined with AZD4635 in the MCA205 model (**Figure 1C**; and **Supplementary Figure S4**). Interestingly, higher efficacy of monotherapies and combinations was observed in studies using the MCA205 syngeneic model (*vs.* CT26 and MC38 studies). Dose-dependent decreases in tumor growth rates were found in an additional MCA205 study, for an AZD4635 dose range of 10–50 mg/kg, in combination with the anti-PD-L1 mAb, although confidence intervals overlapped among these groups (see **Figure 1C**; and **Supplementary Materials, Figure S6**).

To evaluate the adequacy of TME representation in the QSP model, we performed model qualification runs using flow cytometry data collected from MCA205 experiments. Various markers were used in the experimental setting, to characterize efficiency of antigen presentation in vehicle- and AZD4635-treated animals, including MHCII and CD86 expressions on macrophages and dendritic cells. This information was generated in MC38 but not in MCA205 experiments; also, the only measurement timepoint was on day 14, which limited our validation exercise. Treatment with AZD4635 was associated with 1.4 to 3.5-fold increases in these markers, while the QSP model predicted a 1.15-fold (90% CI: 0.82–1.91-fold) increase in APC efficiency under such treatment. The model-predicted dTeff level on treatment day 14 was compared to the PD-1-positive CD8+ cell count measured in the experiments. Model simulations and experimental data concurred by indicating a highest increase in cell count under combination treatment, followed by the anti-PD-L1 mAb monotherapy and AZD4635 monotherapy (**Supplementary Materials, Figure S7**).

### Mechanistic Differences Across TMEs in Various Syngeneic Tumor Models

**Figure 2:**
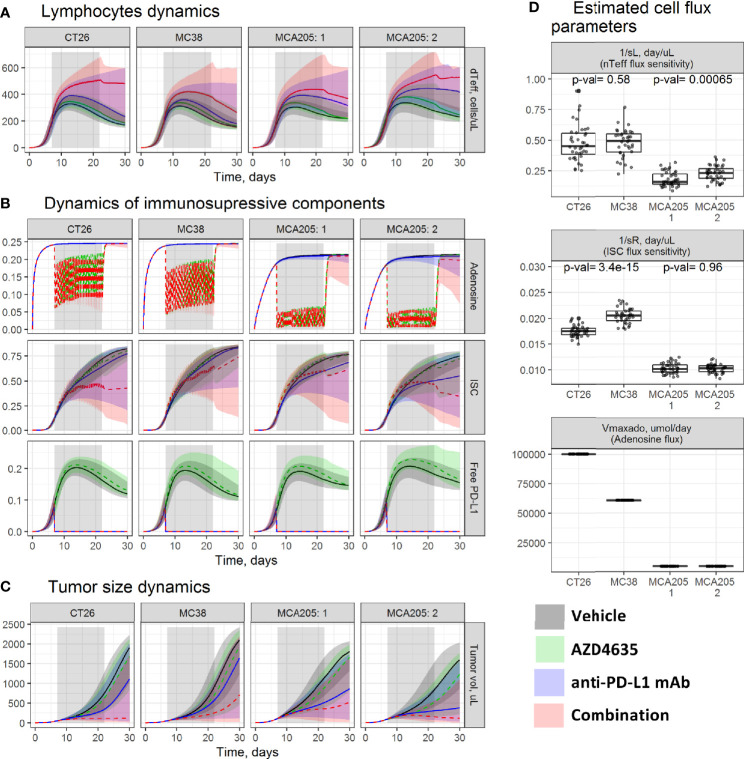
Evaluation of tumor microenvironment (TME) dynamics across experiments. Model-based simulations of **(A)** dTeff; **(B)** key immunosuppressive TME components; and **(C)** tumor size dynamics across studies. Lines—median prediction, area—90% prediction interval (PI). Different treatment regimens are shown by color. **(D)** Boxplots of the estimated parameters across studies. P-values were calculated using the Wilcoxon test. MCA205 experiments: 1—an efficacy study; 2—a dose-finding study.

To identify TME factors responsible for the observed differences in responses across syngeneic models and to investigate mechanisms of AZD4635 and an anti-PD-L1 mAb combination efficacy, we compared parameter estimates across studies and simulated TME dynamics under vehicle, an anti-PD-L1 mAb, AZD4635, or their combination (**Figure 2**).

Model simulations indicated similarity in TME dynamic trends across studies. In a vehicle group, a transient dTeff increase was observed for the first ~10 days (**Figure 2A**), followed by intratumoral accumulation of multiple immunosuppressive components such as adenosine, ISC, and PD-L1 (**Figure 2B**), which downregulate the dTeff function—a reflection of reduction in intratumoral dTeff level and PD-(L)1 pathway activity. These components can be blocked by the respective therapeutic options; an anti-PD-L1 mAb would enable complete inhibition of PD-L1-mediated effects, whereas AZD4635 would partially inhibit A_2A_R-mediated immunosuppression. These interventions can stimulate dTeff proliferation, with the highest dTeff expansion under combination treatment, which, in turn, would translate into higher efficacy for combinations *vs*. monotherapies.

As can be seen in **Figure 2C**, treatment effects on the TME and, consequently, tumor size dynamics differed across studies. Median tumor growth was slowest under monotherapy and combination treatments in studies performed using MCA205, followed by CT26 and MC38 (**Figure 2C**). The observed BSV in treatment efficacy was captured by applying covariates to four model parameters representing the amount of inoculated tumor cells (TVtot_in_), maximal intratumoral adenosine level (Vmax_Ado_ ), and influxes of dTeff (sL) and ISC (sR). Parameter TVtot_in_ was set based on the experimental protocol (see *Materials and Methods* section). Parameter Vmax_Ado_ was fixed to 100, 60, and 5 µM for, respectively, CT26, MC38, and MCA205, based on intratumoral adenosine measurements (27), resulting in differing target modulation by AZD4635 across these syngeneic models. More pronounced inhibition of the A_2A_R pathway was thus gained in MCA205 *vs*. CT26 and MC38 (**Figure 2B**), which can be explained by more effective competition between AZD4635 and adenosine for A_2A_R. Parameters sL and sR were the main drivers for both BAV and BSV, based on covariate search results; values for these parameters thus differed across animals and studies (**Supplementary Materials, Table S1**). The MCA205 model was characterized by significantly slower dTeff and ISC infiltration levels *vs*. CT26 and MC38, resulting in corresponding lower efficacy of the tested therapies (**Figure 2D**). BSV in treatment efficacy, which was observed between the two MCA205 studies, was explained by differences in parameter sL, with a net result of a more stable dTeff increase and an improved resolution of ISC-mediated immunosuppression.

### Mechanisms of Resistance to the AZD4635 and PD-(L)1 mAb Combination Treatment

**Figure 3:**
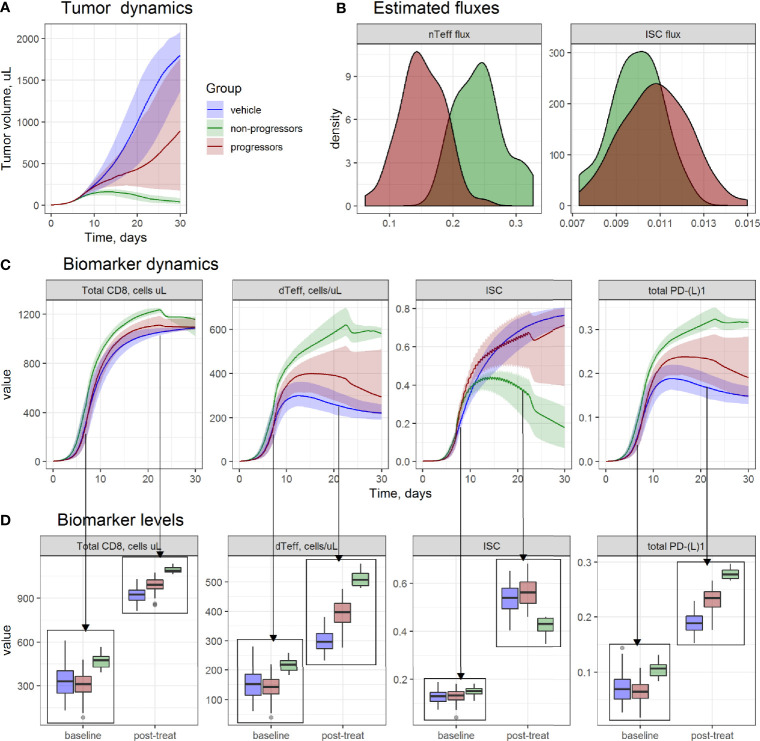
Evaluation of tumor microenvironment (TME) differences between progressors (red), non-progressors (green), and animals from vehicle group (blue), from MCA205 study 1. **(A)** Simulated tumor size dynamics. Lines—median prediction, area—90% prediction interval (PI). **(B)** Density of simulated model parameters. **(C)** Simulated dynamics; and **(D)** boxplots of TME components at baseline and on day 14^th^ day of treatment.

Complete tumor regression under given therapeutic regimens was achieved in a subset of animals only, although, overall, a combination of AZD4635 and an anti-PD-L1 mAb demonstrated highest efficacy across studies (**Figures 1B, C**). Hence, the model was used to investigate TME differences for “progressors” (animals with an increase in tumor size) *vs*. “non-progressors” (animals with tumor size regression or control) during the treatment period, and whether selected TME components would be predictive of response to treatment. To this end, 100 values for parameters **sL** and **sR** were randomly generated from the estimated distributions for the study 1 on MCA205 model and used for simulations of tumor size and TME dynamics. Based on simulated tumor size dynamics, animals were grouped into progressors *vs*. non-progressors, and corresponding changes in each TME model component in each animal were compared, before and after 2 weeks of treatment (**Figure 3**).

Non-progressors were characterized by higher dTeff and lower ISC infiltration rates (**Figure 3B**) in the model, which was reflected in TME dynamics (**Figures 3C, D**). At baseline, non-progressors were characterized by higher baseline dTeff, total CD8 and PD-L1 *vs*. progressors, whereas no significant difference in ISC between non-progressors *vs*. progressors was observed.

A build-up, with time, of both immunoactive and immunosuppressive TME components was observed for all groups, with highest increases in dTeff, total CD8 and total PD-(L)1 for the non-progressors; in contrast, accumulation of ISC in this group was lower *vs*. progressors and vehicle groups. Progressors were characterized by the highest level of ISC after treatment start, whereas increase in immunoactive TME components in this group was lower than in non-progressors but higher than in vehicle-treated animals.

### Evaluation of Potential AZD4635 Combination Strategies

**Figure 4:**
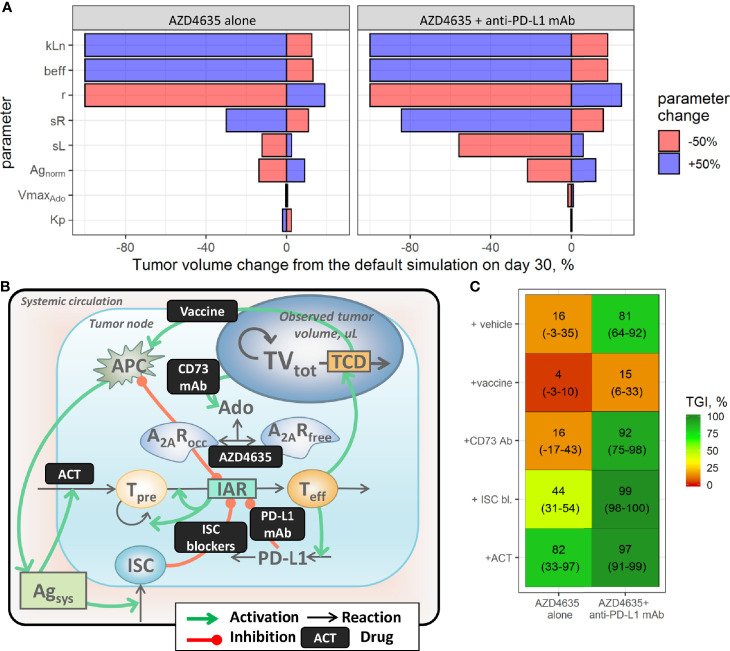
Evaluation of combination strategies for AZD4635. **(A)** Identification of factors affecting the efficacy of AZD4635 alone or in combination with a PD-L1 mAb; colors denote parameter change. **(B)** Implementation of considered treatment options in the model. **(C)** Predicted tumor growth inhibition index (TGI; mean and 90% confidence intervals); colors denote median TGI.

Numerous immunotherapy-based combinations are currently under investigation. However, it would be impractical to test outcomes of all possible treatment combinations, even in the preclinical setting. In this context, a QSP model may be used as a quantitative simulation tool to explore dynamically biomarker responses and efficacy potential of various combinations of modalities, given various TME conditions and modality pharmacokinetics. To provide a rationale for various combinations, we first identified factors which may increase efficacy of AZD4635 alone or in combination with an anti-PD-L1 mAb, *via* a model-based sensitivity analysis. To this end, estimated population parameter values in the most treatment-resistant mouse model (MC38) were varied by ±50%, one by one, and corresponding tumor size responses under AZD4635 alone or in combination with anti-PD-L1 mAb were simulated for a 30-day period. The percentage change from the prediction, obtained using the estimated population parameter values, was calculated and visualized (**Figure 4A**). Parameters affecting maximal nTeff influx (kLn), dTeff cytotoxic activity (beff), and the intrinsic tumor growth rate (r) exhibited a maximal impact on the efficacy of both AZD4365 alone and in combination with the anti-PD-L1 mAb (**Figure 4A**). In contrast, parameters characterizing normalized antigen level (Ag_norm_) as well as antigen-dependent nTeff and ISC influxes (respectively, sL and sR) significantly modulated activity of the combination treatment but had a moderate effect on AZD4635 monotherapy efficacy. Parameters characterizing activation of adenosine and PD-L1-mediated pathways had a minor effect on the TME and tumor dynamics under both monotherapy and combination treatments. To further investigate these dynamics in TME changes under various treatments, we visualized the dynamics of tumor growth, dTeff, ISC, and adenosine-mediated suppression following the systematic variation of parameter values, as described above (**Supplementary Materials, Figure S6**). The most effective treatment options were associated with the highest increase in dTeff and decrease in ISC. Interestingly, a decrease in adenosine production of as much as 50% was followed by a moderate effect on adenosine-mediated immunosuppression under treatment. Decreased tumor growth rates were associated with slower adenosine and ISC build-ups.

While the above mentioned sensitivity analysis represents a mathematical means to model behavior analysis, simulations of preclinical experiments may also provide a sensible representation of various monotherapy and combination scenarios, with predictions of responses to treatment, and enable a systematic evaluation of uncertainty in the observations due to BAV. Therefore, potential combination benefits of AZD4635 or AZD4635/an anti-PD-L1 mAb with other therapies were evaluated *via* virtual preclinical study simulations of the MC38 syngeneic model. To introduce these additional therapies in the QSP model, a scaling factor scF was applied to model parameters, at a timepoint of ≥7 days post tumor inoculation, to mimic various processes modulation by the treatment (**Figures 4A, B**). Selection of scF parameter values was challenged by insufficient quantitative information, as generated for particular immunotherapeutic interventions; therefore, value selection was based upon certain assumptions. To simulate dosing of CD73 mAb in the model, the parameter reflecting the adenosine synthesis rate (Vmax_Ado_) was set to zero, assuming complete blockade of CD73-mediated adenosine production under treatment. The efficacy of compounds targeting adenosine/PD-L1-independent immunosuppressive mechanisms (*e.g.*, CTLA-4 mAb) was captured by setting the ISC flux parameter (sR) to zero, to reflect complete blockade of these pathways. Adoptive cell transfer (ACT) was mimicked *via* a 2-fold increase in the nTeff influx parameter (sL), to reflect the increase in tumor-infiltrating lymphocytes following the intervention. The efficacy of vaccines was investigated *via* a 2-fold increase in the antigen flux parameter (Ag_norm_)—acknowledging that no experimental information on *in vivo* levels of the presented tumor antigen was found in the literature.

Virtual experiments with 10 animals *per* group were simulated using 10 pairs of randomly generated values for parameters sL and sR. A tumor growth inhibition index (TGI) on day 28 was used as an efficacy outcome (Equation 1):

(1) $$TGI=\left(1-\frac{GeoMean\left(TV_{tr}\right)}{GeoMean\left(TV_{con}\right)}\right)\cdot 100\%$$

where GeoMean(TV_tr_) and GeoMean(TV_con_) are geometric means of tumor volumes in the treated and control groups, measured 2 weeks after start of treatment.

To evaluate between-study uncertainty in the TGI estimates, which arises from the relatively low number of animals used, the simulations were repeated 100 times, median and 95% confidence intervals for TGI were calculated; results of these simulations are summarized in **Figure 4C**.

Various therapeutic modalities added on top of AZD4635 alone or in combination with an anti-PD-L1 mAb differentially modulated anti-tumor activity of the treatments. Blockade of adenosine production with an CD73 mAb was followed by decreases in adenosine levels leading to improvement of A_2A_R coverage by AZD4635. This effect was associated with increase of anti-tumor activity of AZD4635 and an anti-PD-L1 mAb in combination, in contrast, efficacy of the dual combination of AZD4635 and CD73 mAb was limited by activation of PD-L1 and ISC pathways (**Supplementary Figure S8**). Downregulation of ISC influx would significantly improve the efficacy of both mono- and combination therapies, indicating a high therapeutic potential of AZD4635 combinations with various therapeutic approaches which target immunosuppressive mechanisms (*e.g.*, CTLA-4 mAb). The addition of a vaccine to treatment was predicted to be less effective *vs*. other options, which may be explained by the activation of tumor infiltration not only by immunoactive cells but also by immunosuppressive cells, with a subsequent downregulation of effector T cell clonal expansion (**Supplementary Figure S8**). Model simulations suggested ACT to be an effective modality to combine with AZD4635, due to a pronounced and stable increase in effector T cells under this combination treatment.

## Discussion

Several quantitative models have been proposed to investigate mechanisms of drug action and to support drug development programs in immuno-oncology (IO) (28). The granularity of mechanistic details incorporated into the model structure depends on study objectives, experimental data availability, and current knowledge in the field, which have led to various mathematical representations of biological mechanisms. In the present study, we built upon a QSP model previously developed by Kosinsky et al. (21). This model captured key steps of the cancer immunity cycle and enabled a ‘condensed’ representation of the immunosuppressive components of the TME, as a lumped function of molecular and cellular events affected by treatment(s) (*e.g.*, PD-L1- and A_2A_R-mediated pathways) or not explicitly modulated by treatment(s) (*e.g.*, presence of Tregs). This parsimonious approach to QSP model development allowed us to reproduce and quantitatively characterize well-established IO “categories,” such as adaptive immune tolerance, tumor immunogenicity and immunogenic tumor cell death (TCD) (29) and, at the same time, to minimize uncertainty in model parameter estimation—a result of the biological complexity stemming out of numerous dynamic interactions within the TME (28).

With this parsimonious approach, several assumptions on adenosine biology were made during the model development process. Firstly, A_2B_R-related mechanisms were not included in the model, given contradictory information in the literature on the role of A_2B_R in immunosuppression. Given the relatively low affinity for adenosine to A_2B_R (Kd ~ 10–100 µM) and the ~ 0.1–1 nM range of extracellular adenosine in the TME (1, 2), A_2B_R would not be expected to significantly contribute to adenosine-mediated immunosuppression. Also, experiments by Kjaergaard and colleagues have shown that A_2B_R knock-out does not significantly affect the growth of MCA205 tumors (30). Interestingly, in contrast to these observations, anti-tumor activity has been detected for several selective A_2B_R inhibitors in 4T1 and B16.F10 syngeneic models (12, 13, 31). Secondly, heterogeneity in spatial adenosine distribution within the TME (17) and detailed adenosine transport steps between intra- and extra-cellular compartments (2, 17, 32) were considered as out of scope for the present modeling research. To account for differences between measured total TME (intra- and extra-cellular) adenosine concentrations and effective adenosine concentrations affecting immune cells, a correction factor Ado_scF_ was applied and the effect of different values of Ado_scF_ was tested *via* a sensitivity analysis.

The QSP model was used to gain a further understanding of AZD4635 effects on the TME and, consequently, on tumor size dynamics. Although the proposed model is not designed to provide insights into actual molecular mechanisms of experimental observations and does not differentiate among various immunosuppressive mechanisms (*e.g.*, myeloid-derived suppressive cells and tumor-associated macrophages), it can be used to track temporal TME dynamics, which cannot be achieved in the experimental setting. In *in vivo* experiments, TME modulation by various treatments is typically evaluated through tumor tissue flow cytometry data; such measurements represent only “snap-shots” of the TME and do not provide information on dynamic changes which do occur during the tumor evolution process, while a particular treatment gets periodically delivered *via* dosing of the therapeutic modality (33). Our model-based simulations indicate primary activation of the immune system, with a secondary tumor-driven influx of immunosuppressive components (ISC) as well as triggering intrinsic factors that limit anti-tumor efficacy of the immune response (*e.g.*, PD-(L)1), in all three syngeneic models tested (CT26, MC38, MCA205). These TME simulations are in line with experimental observations reported by Lechner and colleagues in syngeneic tumor models with a “hot” immunophenotype, which is characterized by a build-up of both pro- and anti-inflammatory cells during tumor progression (34–36).

The combination of AZD4635 and an anti-PD-L1 agent is thought to potentially shift the TME balance from an anti- to a more pro-inflammatory state, to then enable more complete tumor regressions—although treatment resistance has been observed in some animals. We captured these features in the QSP model, through variability in fluxes of immunosuppressive and immunoactive cells, in line with concepts of innate and adaptive immune tolerance (29). These findings underline the importance of a high tumor infiltration by effector T cells and predominant contribution of adenosine-related pathway into immunosuppression, which has been highlighted in a recent publications by Dr. Sitkovsky (37, 38). From a clinical perspective, these observations provide a strong rationale for measuring tumor infiltrating lymphocyte densities (*e.g., via* an Immunoscore test) (39) or intratumoral PD-(L)1 expression. Interestingly, our *in vivo* simulations indicated higher tumor infiltration by dTeff in non-progressors *vs.* progressors, in both baseline and post-treatment state; in contrast, differences in the activity of immunosuppressive components in the TME between these two groups (non-progressors *vs*. progressors) were manifest only at the end of treatment. This could be explained by a later activation of ISC *vs.* dTeff influx into the tumor, as reflected in higher values of sR *vs.* sL parameters which characterize the respective cell fluxes – this may represent, in fact, the mechanism of acquired resistance, causing disease progression after an initial response to treatment.

Variability in the sL and sR parameters also drove differences in AZD4635 and anti-PD-L1 mAb combination efficacy across different syngeneic models, in line with preclinical findings by Mosely (35), Lechner (34), and Yu (40). Experimental observations indicate higher AZD4635 activity in CT26 *vs*. MC38; similar results were obtained for the adenosine inhibitor CPI-444 (17, 32). In the QSP model, these findings were explained by a higher infiltration of ISC, in MC38 *vs.* CT26. This model-based observation was supported by a higher frequency of mMDSC in MC38, observed experimentally in preclinical studies (35). Intratumoral adenosine levels represent another source of BSV; in the QSP model, these were set at a ~10-15 fold higher in MC38 and CT26 *vs*. MCA205, in accordance with experimental measurements (17). Based on the competitive inhibition mechanism represented in the QSP model, higher adenosine levels were associated with lower A_2A_R blockade by AZD4635, which may also contribute to lower treatment efficacy, as observed in the CT26 and MC38 studies. Intuitively, a decrease in intratumoral adenosine levels *via* targeting of the ATP metabolic pathway, *e.g.*, with CD73- or CD39-specific antibodies may show additional benefits compared to AZD4635 monotherapy, as illustrated by model-based simulations as well as preclinical data for another A_2A_R inhibitor, SCH58261 (8). As intratumoral hypoxia was shown to be an important factor regulating CD39 and CD73 expression, targeting upstream hypoxia-HIF-1 factor may be also used as an effective strategy to decrease adenosine production (3, 4).

Another advantage in combining inhibitors of A_2A_R and CD73 may reside in the potential decrease of A_2B_R occupancy, even though contribution of these receptors toward immunosuppression may not be too significant, given contradicting experimental data as discussed above. Meanwhile, co-inhibition of A_2A_R and CD73 has been shown to be more effective *vs*. CD73 blockade alone (41). This may be explained by potential additional A_2A_R activation *via* AMP, which may build up under CD73 inhibition conditions (42); however, given the ~100-fold lower affinity of AMP *vs.* adenosine for A_2A_R, this hypothesis would require further investigation.

To identify further strategies for improvement of AZD4635 efficacy in progressors (non-responsive animals), we first performed a model-based sensitivity analysis, which helped us identify factors affecting treatment outcomes. Next, we evaluated, *via* preclinical study simulations, various therapeutic options which would modulate corresponding pathways. For example, an increase in Teff levels was predicted to significantly improve the efficacy of AZD4635 alone or in combination with an anti-PD-L1 mAb; this provides a rationale for combination of AZD4635 with, for example, ACT, and was supported by our model-based preclinical study simulations as well as recent preclinical experimental evidence (37). Interestingly, stimulation of antigen presentation in our QSP model did not result in an increase in AZD4635 and an anti-PD-L1 mAb combination efficacy in MC38-like tumors, presumably due blockade of the initial immune response by immunosuppressive components as predicted by the model (**Supplementary Figure 6**). These observations could explain high attrition rates observed in some early clinical immunotherapeutic interventions, implying non-specific activation of the immune system such as IL-2 (43). However, when interpreting such observations, multiple factors (*e.g.*, vaccine immunogenicity, ACT technology) need to be taken into account, to provide a more accurate mechanistic description of treatment efficacy.

While simultaneous targeting the adenosine synthesis and signaling pathways with various treatments (*e.g.*, CD73 and A_2A_R inhibition) may result in incremental efficacy benefits, as discussed above, our model-based simulations suggest that multi-pronged targeting of different TME components to be the most effective treatment strategy; this has also been emphasized in a recent publication (37). These results are further supported by recent preclinical evidence: efficacy of adenosine-targeting compounds (CD73 or an A_2A_R inhibitors) was significantly increased in combination with an anti-CTLA-4 mAb, an anti-PD-1/L1 mAb, or DC vaccine (27, 44, 45).

Whereas the current modeling work is based solely on preclinical knowledge and experimental data available for the analysis, a number of limitations should be noted; also, additional experimental data would allow for further improvements to the proposed QSP model. For example, an investigation in a clinical setting, of patient-to-patient variability in tumor dynamics behavior, would help to identify predictive biomarkers associated with treatment response—to then be used toward patient selection in further trials. Main factors which may limit the use of mechanistic QSP NLME models include a lack of immunohistochemistry and flow cytometry data, to track treatment-induced changes in the TME, and an insufficient number of available biopsy samples from early phase studies, to calibrate statistical components of the models. Given these uncertainties, model structure should be adapted accordingly, to avoid the use of unrealistic assumptions and fixing model parameters with non-physiological values. While our QSP modeling approach is more of a “hypothesis-driven” approach—with the model structure being dictated by *a priori* knowledge, “data-driven” multivariate machine learning approaches can be used to gain more specific information on molecular mechanisms, from clinical trial observations. Two combinatorial omics-based adenosine-related signatures, associated with clinical response to A_2A_R inhibitors, have been proposed by Sidders et al. (20) and Fong et al. (19). Both publications indicate higher treatment efficacy in patients with pronounced activation of the adenosine-mediated pathway, pointing again to the importance of a high contribution of this mechanism toward the overall immunosuppression balance. To further maximize the use of -omics data and account for dynamic changes in the anti-tumor immune response under therapeutic treatment, the hybrid approach, which exploits biomarker signatures (derived out of data-driven machine learning methods) as input functions to ODE-based NLME models, holds great potential to improve upon model-based predictive simulations (28).

## Conclusion

The proposed quantitative modeling approach enabled the integration of diverse preclinical information, to investigate TME-mediated mechanisms of response and of resistance to AZD4635-based therapies, and provided a rational simulation framework to test various combination strategies, with a goal of improving the probability of success in treatment efficacy.

## Data Availability Statement

The original contributions presented in the study are included in the article/**Supplementary Material**. Further inquiries can be directed to the corresponding author.

## Author Contributions

Conceptualization: YK, KP, VV, KS, and GH. Methodology: VV, LC, YK, KP, HK, and GH. Formal analysis: VV, LC, GH, KP, and YK. Investigation: VV, LC, KP, GH, KS, WS, SB, and MM. Writing–original draft: VV, KP, and GH. Writing–review and editing: VV, LC, KP. Visualization: KP and VV. Supervision: KP, GM, HK, RK, WS, and GH. All authors contributed to the article and approved the submitted version.

## Funding

This work was funded by AstraZeneca Pharmaceuticals. The funder bodies were not involved in the study design, collection, analysis, interpretation of data, the writing of this article or the decision to submit it for publication.

## Conflict of Interest

GH, LC, AB, RW, KS, WS, RK, GP, MM, HK and GM are employees of AstraZeneca; VV, YK and KP are employed by M&S Decisions LLC.

The remaining authors declare that the research was conducted in the absence of any commercial or financial relationships that could be construed as a potential conflict of interest.

## Abbreviations

A_2A(B)_R, adenosine receptor type 2A(B); ACT, adoptive cell transfer; BAV, between-animal variability; BSV, between-study variability; CTLA-4, cytotoxic T-lymphocyte-associated protein 4; DC, dendritic cells; ISC, immunosuppressive cells; IO, immuno-oncology; NLME, non-linear mixed-effects; mAb, monoclonal antibody; Teff, effector T cells; TME, tumor microenvironment; OFV, objective function value; PD-L1, Programmed death-ligand 1; PK, pharmacokinetic; TCD, tumor cell death; TGI, tumor growth inhibition; QSP, quantitative systems pharmacology.
