# Supplementary material for: Evaluation of Combination Strategies for the A2AR Inhibitor AZD4635 Across Tumor Microenvironment Conditions via a Systems Pharmacology Model
Source: Front Immunol. 2021 Mar 2;12:617316. doi: 10.3389/fimmu.2021.617316 (PMC7962275; doi:10.3389/fimmu.2021.617316)
Supplement: Supplementary file 1 [file DataSheet_1.docx]

Supplementary Material to the manuscript:

***Evaluation of combination strategies for the A_2A_R inhibitor AZD4635 across tumor microenvironment conditions via a systems pharmacology model***

The proposed model was developed in a step-wise manner; publicly available and internal data were used to inform model structure and parameters (Figure 1); key steps in model development are detailed in further sections below.


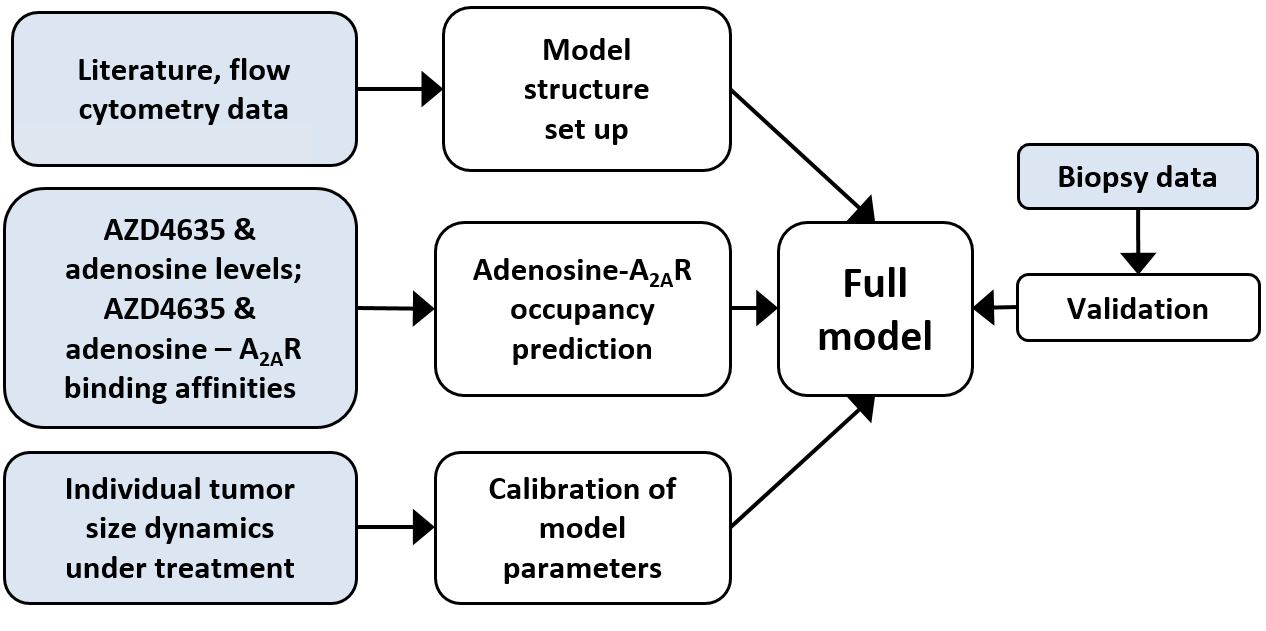


***Figure S1.*** *Model development flow chart*

# Biological rationale for the proposed model structure

The fundamental structure of the proposed model is based on a quantitative systems pharmacology (QSP) model of the cancer immunity cycle in immuno-oncology (IO), which we published previously and which provides further information *vs*. specific details highlighted here (1):

## IO core model structure based on cancer immunity cycle concept

1. Both innate and adaptive immune system are involved in anti-tumor immune response(2). Whereas innate immunity, represented by NK cells, controls immune surveillance, fighting off tumor initiation and the metastatic process(2–4), adaptive immunity affects later stages of tumorigenesis by constraining tumor growth (4). The current modeling work is focused on the dynamics of tumor size and, therefore, considers the adaptive immune response only.
2. Anti-tumor immune response may be described according to the cancer immunity cycle concept proposed by Chen *et al*. (5). The cycle is triggered by antigen presentation, which is performed by mature antigen presenting cells (macrophages and dendritic cells) expressing co-stimulatory molecules (*e.g.* CD86). The process is followed by infiltration of antigen-specific T cell clones into tumor tissue, with subsequent proliferation. Such clones are able to recognize and kill tumor cells specifically. This process results in additional tumor antigen release, allowing re-initiation of the cycle (5).
3. Distinct molecular mechanisms affecting different stages of the cycle are exploited by tumors, to escape immune response. Various populations of immunosuppressive cells (ISC) such as Tregs and gMDSCs(6,7), other immune checkpoints such as Tim3 and Lag3(8), and metabolites such as nitric oxide (9) may downregulate anti-tumor immune response. The present modeling work was focused on the investigation of adenosine and PD-L1-mediated immunosuppression; hence, corresponding biological knowledge captured in the model is further detailed here.
4. Several preclinical studies have shown that PD-1/PD-L1 interactions suppress T cell proliferation, pro-inflammatory cytokine production, and antigen-dependent cytotoxicity (10). Release of interferon-gamma (IFNy) by activated immune cells represents one of the key drivers for PD-L1 expression. IFNy induces tumor cell PD-L1 expression *in vitro* in a dose-dependent manner, with a characteristic time of about 24 hours (11).

## Role of adenosine in immunosuppression

1. Adenosine is a product of step-wise ATP dephosphorylation, catalyzed by CD39 (ATP conversion to ADP) and CD73 (ATP conversion to adenosine (12). Several factors, including (1) release of adenosine and its precursors, from dying tumor cells, into the extracellular space (12,13); (2) activation of enzymes involved in adenosine production *via* hypoxia and other factors (*e.g.,* as triggered by particular therapeutic interventions) (14–17); (3) inhibition of enzymes involved in adenosine conversion to AMP (adenosine kinase) (15) may all result in abnormal adenosine build-up within tumor tissue (18).
2. Total (intra- and extra-cellular) adenosine concentration was shown to be similar across tumor sizes, in a range of 100-800 μL (16,17). We thus assumed that adenosine levels would reach a steady state in relatively small tumors <100 μL (19). Total adenosine concentration ranges from 5 to 150 µM depending on the syngeneic mouse model used; furthermore, extracellular adenosine levels are ~10-fold lower *vs*. intracellular ones (19,20). In addition, adenosine is spatially heterogeneously distributed within tumor tissue, which further affects a precise estimation of adenosine levels modulating the activity of immune cells (19).
3. Adenosine may act as a regulatory force in the TME *via* multiple mechanisms, through the activation of A_2A_R and A_2B_R receptors (21). The affinity of adenosine for A_2A_R is much higher than for A_2B_R (EC_50_ values of, respectively, 10^-7^-10^-6^ *vs*. 10^-5^-10^-4^ M) (22) and hence, the role of A_2B_R in the TME of these various syngeneic models is unclear. Current work by Kjaergaard and colleagues showed that MCA205 tumor growth was delayed in A_2A_R knockout mice, whereas A_2B_R knockout did not affect tumor size dynamics (23); this may be explained by relatively low adenosine levels in MCA205, insufficient to activate A_2B_R. In some preclinical studies of B16 melanoma, A_2B_R blockade was shown to inhibit tumor growth, which points to the importance of A_2B_R activation in adenosine-mediated immunosuppression (24,25); however, there have been no studies, to date, demonstrating efficacy of selective A_2B_R inhibitors in CT26 or MC38 syngeneic tumors.
4. Adenosine may decrease the efficiency of antigen presentation, presumably *via* both A_2A_R and A_2B_R: (1) *in vitro* exposure of human monocytes to 5′-(N-ethylcarboxamido) adenosine (NECA) (a stable adenosine analogue, non-selective A_2A_R and A_2B_R agonist) resulted in the formation of functionally impaired DC with pro-angiogenic and anti-inflammatory properties; this effect has not been observed in A_2B_R-knockout monocytes (26,27); (2) treatment of MC38-bearing mice with AZD4635 was followed by an increased expression of co-stimulatory markers (CD86) and markers of antigen presentation (MHC II) on tumor infiltrating DC and macrophages (19). Adenosine has also been shown to directly suppress NK and lymphocyte activity and promote their exhaustion *via* A_2A_R (21). In addition, adenosine may activate other immunosuppressive mechanisms: A_2A_R activation is associated with Treg induction (21), whereas A_2B_R-mediated immunosuppression involves gMDSC accumulation (28). Exposure of T cells to high adenosine concentrations (10 µM) has been shown to fully block their functional activation and proliferation (29,30).

## Variability in TME and treatment efficacy across syngeneic tumor models

To evaluate TME differences across various syngeneic tumor models, flow cytometry measurements from publicly available sources were reviewed. Differences in gating strategy and other technical features across studies rendered a direct comparison of observations from multiple experiments challenging; hence, we focused on the more exhaustive studies by Mosely, Lencher and Yu groups, conducted in several syngeneic models (6,7,31). Key findings from the papers are summarized here:

1. Some tumors are not immunogenic and are poorly infiltrated by immune cells (*e.g.,* B16), whereas other tumors are characterized by various types of immunosuppressive cells including Tregs (CT26 and RENCA), gMDSC (4T1), or mMDSC (CT26, MC38 and LL2).
2. Dynamical changes in TME differ among syngeneic models: over time, CD8, Treg and DC tend to increase in 4T1 and CT26, as tumors grow from ~50 to 900 µL. MDSC accumulation is observed in 4T1, B16 and RENCA, whereas moderate MDSC reduction is observed in CT26;, no time-dependent dynamic data are available for MC38 and MCA205 (7).
3. Tumor growth rates have been shown to correlate with tumor immunogenicity. For example, poorly immunogenic B16 tumors exhibit the fastest growth rate (6), which indicates that the observed differences in tumor size dynamics across experimental models can be driven by TME properties and not only by intrinsic tumor cell growth rates.
4. Adenosine levels also vary across syngeneic tumor models. The total (intra- and extra-cellular) adenosine concentration can range from ~5 μM (MCA205) to ~100 μM (CT26) (19).
5. Anti-tumor efficacy of IO treatments depends on TME properties: low efficacy of DC vaccines, anti-PD-L1 and anti CTLA4 mAb has been found in poorly immunogenic B16 tumors (6,7). Responses of highly immunogenic tumors differed across syngeneic models. For example, CT26 and MC205 have been shown to be responsive to PD-L1 mAbs, whereas RENCA and 4T1 did not respond to such treatment (7); MC38 tumor growth was inhibited by an anti-PD-L1 mAb in one study (32), whereas in another study, the effect of an anti-PD-L1 mAb on MC38 growth was not significant (7). The growth of CT26 and RENCA was inhibited by an anti-CTLA-4 mAb, which was not the case for MC38 and 4T1 (7). AZD4635 inhibited tumor growth in CT26, MC38 and MCA205, however, treatment efficacy differed across studies (32).

# Structural model

The above key features were selected to dictate the key molecular and cellular players in the cancer immunity cycle to be included in the QSP model and to capture mechanisms of drug action. In this Section, we describe all model equations used. Some of these have been taken from our previously published work by Kosinsky *et al*. (1), as specified in the text where appropriate.

## AZD4635 and PD-(L)1 pharmacokinetic models

A one-compartment pharmacokinetic (PK) model with first-order absorption and linear elimination, developed previously (1), was used to reproduce the drug concentration profile of the anti-PD-L1 mAb (1) (Equations 1-2):

$\frac{\mathrm{dmA}b_{\mathrm{ad}}}{\mathrm{dt}}=-kabs_{\mathrm{mAb}}\cdot mAb_{\mathrm{ad}}$ (1)

$\frac{\mathrm{dmA}b_{c}}{\mathrm{dt}}=kabs_{\mathrm{mAb}}\cdot mAb_{\mathrm{ad}}-kel_{\mathrm{mAb}}\cdot mAb_{c}$ (2)

Where $\mathrm{mA}b_{\mathrm{ip}}$ and $\mathrm{mA}b_{c}$ are amounts of the anti-PD-L1 mAb in the administration and central compartments.

A two-compartment PK model with extravascular drug administration was set to characterize AZD4635 concentrations - a PK model structure which best described the experimental data (Equations 3-5):

$\frac{d\mathrm{AZD}_{\mathrm{ad}}}{\mathrm{dt}}=-\frac{\mathrm{Vmaxab}s_{\mathrm{AZD}}\cdot\mathrm{AZD}_{\mathrm{ad}}}{EC50abs_{\mathrm{AZD}}+\mathrm{AZD}_{\mathrm{ad}}}$ (3)

$\frac{d\mathrm{AZD}_{c}}{\mathrm{dt}}=\frac{\mathrm{Vmaxab}s_{\mathrm{AZD}}\cdot\mathrm{AZD}_{\mathrm{ad}}}{EC50abs_{\mathrm{AZD}}+\mathrm{AZD}_{\mathrm{ad}}}-\mathrm{kel}_{\mathrm{AZD}}\cdot\mathrm{AZD}_{c}-(\frac{Q_{\mathrm{AZD}}}{Vc_{\mathrm{AZD}}}\cdot\mathrm{AZD}_{c}-\frac{Q_{\mathrm{AZD}}}{V\mathrm{cp}_{\mathrm{AZD}}}\cdot\mathrm{AZD}_{p})$ (4)

$\frac{d\mathrm{AZD}_{p}}{\mathrm{dt}}=\frac{Q_{\mathrm{AZD}}}{Vc_{\mathrm{AZD}}}\cdot\mathrm{AZD}_{c}-\frac{Q_{\mathrm{AZD}}}{Vp_{\mathrm{AZD}}}\cdot\mathrm{AZD}_{p}$ (5)

Where $\mathrm{AZD}_{\mathrm{ad}}$, $\mathrm{AZD}_{c}$, $\mathrm{AZD}_{p}$ represent AZD4635 amounts in, respectively, administration, central and peripheral compartments; $\mathrm{Vmaxab}s_{\mathrm{AZD}}$ and $EC50abs_{\mathrm{AZD}}$ are, respectively, the maximal absorption rate and the AZD4635 amount in the intestinal compartment associated with half-maximal absorption; $Q_{\mathrm{AZD}}$ is the intercompartmental clearance;$\mathrm{kel}_{\mathrm{AZD}}$ is the AZD4635 elimination constant; and $Vc_{\mathrm{AZD}}$ and $Vp_{\mathrm{AZD}}$ are volumes of, respectively, central and peripheral compartments.

## Description of tumor size dynamics

The intrinsic growth dynamics of tumors (a volume, in mL) was described by a logistic equation, which captures tumor cell death (TCD) as mediated by immune-dependent and other mechanisms, as described in the Kosinsky *et al*. model (1) (Equation 6):

$\frac{\mathrm{dTV}}{\mathrm{dt}}=r\cdot TV\cdot\left( 1-\frac{\mathrm{TV}}{TV_{\max}} \right)-TCD$ (6)

Where TV is the tumor volume; r, TV_max_ are parameters describing the intrinsic tumor growth rate and the upper limit of tumor size, respectively; TCD is a function dependent on tumor-specific mature T cell clones $\mathrm{dTeff}$ (Equation 7):

$TCD=(beff\cdot dTeff+d_{0})\cdot TV$ (7)

Where $beff\cdot dTeff$ represents the tumor cell killing rate, governed by $\mathrm{dTeff}$, with beff as an efficacy coefficient; d_0_ is a slow constant rate representing other processes leading to tumor cell kill.

## Modeling of the cancer immunity cycle

TCD is followed by tumor antigen release into the extracellular space and stimulation of the antigen-presenting process, which was assumed to be downregulated by activated A_2A_R, based on experimental observations (32) (Equation 8):

$\mathrm{Ag}_{\mathrm{sys}}=TCD\cdot\left( 1-A2ARs \right)\cdot Ag_{\mathrm{norm}}$ (8)

Where $\mathrm{Ag}_{\mathrm{sys}}$ represents systemic antigen levels, $A2ARs$ represents the effect of activated A_2A_R on antigen presentation, which will be described further; $Ag_{\mathrm{norm}}$ is a control parameter, used to reflect fold changes in the systemic antigen supply under vaccine treatment

The description of T cell-mediated immune response was taken from the previously developed model (1). Two populations of T cells were considered: (1) non-differentiated and relatively long-lived T cells (nTeff), which may proliferate and differentiate to (2) terminally differentiated, shorter-lived and non-proliferating cytotoxic effector T cells (dTeff). T cell proliferation and differentiation take place within tumor tissue and are regulated by TME properties. The time dynamics of nTeff and dTeff populations are described by Equations 9 and 10:

$\frac{\mathrm{dnTeff}}{\mathrm{dt}}=k_{\mathrm{LN}}\cdot\frac{Ag_{\mathrm{sys}}}{Ag_{\mathrm{sys}}+sL}+IAR\cdot\left( k_{\mathrm{pro}}-k_{\mathrm{dif}} \right)\cdot nTeff-k_{\mathrm{el}}\cdot nTeff$ (9)

$\frac{\mathrm{ddTeff}}{\mathrm{dt}}=IAR\cdot k_{\mathrm{dif}}\cdot nTeff-k_{\mathrm{apo}}\cdot dTeff$ (10)

Where $k_{\mathrm{LN}}$ represents the maximal influx rate of nTeff cells; sL represents the sensitivity of non-differentiated T cell influx that is driven by $Ag_{\mathrm{sys}}$; kpro, kdif, kel and kapo are rate constants of, respectively, T cell proliferation and differentiation, slow and fast elimination of nTeff and dTeff (1)

IAR is an “immune activation rate” lumped semi-empirical function that includes several TME components such as PD-L1, adenosine and different types of immunosuppressive cells (Equation 11):

$IAR=\left( 1-PDL1free \right)\cdot\left( 1-A2ARs \right)\cdot(1-ISC)$ (11)

These TME components are represented by their respective functions $PDL1free$, $A2Ars$ and $\mathrm{ISC}$, with values ranging from 0 to 1 - where 1 denotes maximal immunosuppression and in the absence of any Teff proliferation and activation. A description for each of these functions is given in further sections below.

## Modeling of TME dynamics

The description of PD-L1 dynamics within the tumor was taken from the Kosinsky *et al*. model (1). PD-L1 expression was assumed to be dependent on the level of differentiated active effector cells (Equation 12):

$\frac{dPDL1}{\mathrm{dt}}=k_{PDL1}\cdot(\frac{\mathrm{dTeff}}{dTeff+K_{P}}-PDL1)$ (12)

Where $k_{PDL1}$represents PD-L1 turnover; $K_{P}$is a sensitivity parameter of PD-L1 induced by dTeff.

The immunosuppressive PD-L1 effect depends on free PD-L1 levels and can be reduced by an anti-PD-L1 mAb (Equation 13):

$PDL1free=\frac{PDL1}{1+mAb/Kd}$ (13)

Where mAb is the drug concentration in plasma and $\mathrm{Kd}$ (nM) represents the antibody dissociation constant.

As discussed in previous sections, the distribution of adenosine within tumor tissue is heterogeneous. In scope with the present modeling work and in accordance with NLME methodology principles, a simplified description of adenosine dynamics within the TME was used. The accumulation of adenosine was assumed to be dependent on tumor size, and its elimination was described by a linear equation (Equation 14):

$\frac{\mathrm{dAdo}}{\mathrm{dt}}=\mathrm{kto}_{\mathrm{Ado}}\cdot(\frac{\mathrm{Ad}o_{\mathrm{scF}}\cdot Vmax_{\mathrm{Ado}}\cdot Tum}{\mathrm{EC}{50}_{\mathrm{Ado}}+Tum}-Ado)$ (14)

Where$\mathrm{Ado}$ is the average adenosine concentration in the tumor;$k_{\mathrm{Ado}}$ represents adenosine turnover; $\mathrm{Vma}x_{\mathrm{Ado}}$ is the maximal adenosine production rate, estimated based on total intratumoral adenosine content;$\mathrm{Ad}o_{\mathrm{scF}}$ is a scaling factor enabling conversion of total intratumoral adenosine content to extracellular adenosine levels; $\mathrm{EC}{50}_{\mathrm{Ado}}$ represents the sensitivity of adenosine production to tumor size; and$\mathrm{kto}_{\mathrm{Ado}}$ is an apparent adenosine turnover constant.

The fraction of A_2A_R receptors occupied by adenosine was calculated using Equation 15 (33), which considers competitive reversible A_2A_R inhibition by AZD4365:

$A2AR_{\mathrm{Ado}}=\frac{Ado/\mathrm{Kd}_{\mathrm{Ado}}^{A2AR}}{1+Ado/\mathrm{Kd}_{\mathrm{Ado}}^{A2AR}+\frac{\mathrm{AZD}_{C}/MW_{\mathrm{AZD}}\cdot{10}^{6}}{\mathrm{Kd}_{\mathrm{AZD}}^{A2AR}}}$ (15)

Where $\mathrm{Kd}_{\mathrm{Ado}}^{A2AR}$ and$\mathrm{Kd}_{\mathrm{AZD}}^{A2AR}$ are dissociation constants for, respectively, adenosine and AZD4635 binding *vs*. A_2A_R; and $MW_{\mathrm{AZD}}$ is the AZD4635 molecular weight.

The effect of adenosine-dependent A_2A_R occupancy on the functional activity of dTeff and APC was assumed to be the same and was described using an Emax-type equation (Equation 16):

$A2ARs=\frac{\mathrm{Fmax}_{A2Rs}\cdot A2AR_{\mathrm{Ado}}}{{EC50}_{A2Rs}+A2AR_{\mathrm{Ado}}}$ (16)

Where $\mathrm{Fmax}_{A2Rs}$ is the maximal adenosine effect on dTeff and APC suppression; and${EC50}_{A2Rs}$ is the A_2A_R occupancy associated with a 50% decrease in immune cell activity.

TME components other than PD-L1 and adenosine and which downregulate immune responses were summarized in a variable named ISC; the build-up of ISC is triggered by antigen presentation, mirrors T cell infiltration, and downregulates T cell proliferation and differentiation (1) (Equation 17):

$ISC=\frac{Ag_{\mathrm{sys}}}{Ag_{\mathrm{sys}}+sR}$ (17)

Where sR is a parameter representing the sensitivity of tumor infiltration by immune suppressive cells in response to systemic antigen exposure.

In summary, the proposed model structure provides a minimalistic yet mechanistic description of anti-tumor immune response. As can be seen from Equation 11, immunosuppressive effects of PD-L1 and adenosine are explicitly represented in the model, whereas other TME components, *e.g*., regulatory T cells (Tregs) and B cells (Bregs) are lumped into one variable (ISC).

# Random effects and covariate models

Random effects and covariates were applied to parameters representing intrinsic tumor growth (a), tumor infiltration by T cells (sL, kLn), and TME properties (sR). Values for these model parameters – drivers of between-animal variability (BAV) - were assumed to be different across animals and to be log-normally distributed within the population (34):

$p_{i}=p\cdot exp(ƞ_{i})$ (21)

Where $p_{i}$ reflects the parameter value in the i^th^ animal; and $ƞ_{i}$is a random effect which is normally distributed, with a mean of 0 and a standard deviation of $\Omega_{p}$.

Covariate testing was performed in order to identify factors driving the observed between study variability (BSV) in treatment efficacy:

$p_{j}=exp(\log\left( p_{\mathrm{ref}} \right)+\beta_{j}\left( p \right))$ (22)

Where $p_{j}$ is the population value of the model parameter in the considered group; $p_{\mathrm{ref}}$ is the parameter value in the reference group; and $\beta_{j}\left( p \right)$ is the covariate.

Some of the covariates were fixed based on prior experimental knowledge: parameter $\mathrm{Vma}x_{\mathrm{Ado}}$, which denotes the maximal adenosine accumulation rate, was fixed based on average adenosine estimates from syngeneic models (19); differences in parameter TVin (initial tumor volume) were fixed based on the experimental protocols. Other covariates were applied to one or more model parameters.

Also, several residual error models were tested in the build-out of a TVtot model, and the proportional residual error model was chosen (34):

$\mathrm{TVtot}_{i, j}^{\mathrm{obs}}=\mathrm{TVtot}_{i, j}^{\mathrm{pred}}+(b\cdot\mathrm{TVtot}_{i, j}^{\mathrm{pred}})\cdotɛ_{i,j}$ (23)

Where $\mathrm{TVtot}_{i, j}^{\mathrm{obs}}$ and $\mathrm{TVtot}_{i, j}^{\mathrm{pred}}$ represent the observed and predicted TVtot values, respectively, for animal i at time j; and $ɛ_{i,j}$is an independent random variable normally distributed with a mean of 0 and a standard deviation of 1.

Finally, parameter ScF was fixed at different levels, while other parameters were re-estimated. ScF=1 was selected as a reference, which assumes the adenosine concentration at the site of action is similar to the measured total intratumoral level of adenosine; additional values of ScF < 1 were then tested. A summary of key tested models is given in Table S1.

*Table S1*. Model selection

| Run | ScF | Random effects | Covariates | Identifiability (RSE) | Error model | OFV |
| --- | --- | --- | --- | --- | --- | --- |
| 1 | 1 | r | - | RSE (sL) = 5700% | PROP | 9144 |
| 2 | 1 | kLn | - | RSE (sL) = 13700%  RSE(IC50) = 67% | PROP | 9154 |
| 3 | 1 | sL | - | RSE (IC50) = 71% | PROP | 9170 |
| 4 | 1 | Kp | - | RSE (sL) = 66%  RSE (IC50) = 150%  RSE (sR) = 61%  RSE (kLn) = 69% | PROP | 9149 |
| 5 | 1 | Kado | - | RSE (sL) = 101% | PROP | 9302 |
| 6 | 1 | sR | - | RSE (sL) = 53%  RSE (IC50) = 60% | PROP | 9206 |
| 7 | 1 | sL + sR | - | RSE (IC50) = 165% | PROP | 9140 |
| 8 | 1 | kLn + sR | - | RSE (sL) = 12000%  RSE (sR) = 56%  RSE (IC50) = 55.4% | PROP | 9175 |
| 9 | 1 | sL | sL (study) | + | PROP | 9143 |
| 9 | 1 | sR | sR (study) | RSE (sL) = 84%  RSE (IC50) = 62% | PROP | 9193 |
| 10 | 1 | sL+sR | sL+sR (study) | Beta sR, sL study 2 | PROP | 9072 |
| 11 | 1 | sL+sR | sL (study) +  sR (model) | Beta sR MC38 | PROP | 9067 |
| 12 | 1 | sL+sR | sL (study) +  sR (model) | Beta sR MC38 | CBD 1 | 9082 |
| 13 | 1 | sL+sR | sL (study) +  sR (model) | Beta sR MC38 | CBD 2 | 9076 |
| 14 | 1 | sL+sR | sL (study) +  sR (model) | + | CONST | 9662 |
| 15 | 0.1 | sL+sR | sL (study) +  sR (model) | + | PROP | 9076 |
| 16 | 0.05 | sL+sR | sL (study) +  sR (model) | Beta sR MC38 | PROP | 9078 |
| 17 | 0.001 | sL+sR | sL (study) +  sR (model) | RSE (IC50)=60.6%, Beta sR MC38 | PROP | 9086 |

*PROP, CONST, CBD1,2 – proportional, constant, combined 1, 2 error models, respectively; RSE – residual standard error; NI – not identifiable; OFV – objective function value.*

Table 1 indicates that the incorporation of random effects and covariates into parameters of tumor infiltration by immunosuppressive and immunoactive cells (respectively, sR and sL) allowed for a more accurate and comprehensive reproduction of experimental data, as shown by the drop in OFV. ScF=1 provided the best quality for data reproduction, whereas a decrease in this factor was associated with an OFV increase. Estimated model parameters and diagnostic plots for the selected model are reported and discussed in sections further below.

# Model verification

## Summary of model parameters

Table S2 provides a summary of model parameters.

*Table S2*. Model parameters

| Parameter | Unit | Description | Value | RSE (%) | Source |
| --- | --- | --- | --- | --- | --- |
| **Anti PD-L1 and A_2A_Ri PK and binding parameters** | | | | | |
| **V_d_** | L | Volume of distribution for PD-L1 mAb *i.p.* PKPD model in mouse | 0.003 | - | From (1) |
| **k_a_** | d^-1^ | *i.p.* absorption rate | 8.0 | - | From (1) |
| **kel_mAB_** | d^-1^ | mAb elimination rate | 0.15 | - | From (1) |
| **K_D_** | nM | mAb PD-L1(PD-1) binding affinity | 30 | - | From (1) |
| $\mathbf{Vmaxab}\mathbf{s}_{\mathbf{AZD4635}}$ | mg/d | Maximal AZD4635 absorption rate | 12.6 | 10.6 | Estimated based on PK data |
| $\mathbf{EC50ab}\mathbf{s}_{\mathbf{AZD4635}}$ | mg | AZD4635 amount in intestinal compartment to achieve 50% of maximal absorption rate | 0.178 | 21.9 | Estimated based on PK data |
| $\mathbf{k}\mathbf{el}_{\mathbf{AZD4635}}$ | 1/d | Maximal AZD4635 elimination rate | 320 | - | Fixed |
| $\mathbf{Q}_{\mathbf{AZD4635}}$ | L/d | Intercompartmental clearance for AZD4635 | 9.58 | 18.5 | Estimated based on PK data |
| $\mathbf{Vc}_{\mathbf{AZD4635}}$ | L | Central volume of distribution for AZD4635 | 0.0476 | 34.4 | Estimated based on PK data |
| $\mathbf{Vp}_{\mathbf{AZD4635}}$ | L | Peripheral volume of distribution for AZD4635 | 1.43 | 14.4 | Estimated based on PK data |
| $\mathbf{M}\mathbf{W}_{\mathbf{AZD4635}}$ | g/mol | AZD4635 molecular weight | 315.73 | - | Taken (35) |
| $\mathbf{Kd}_{\mathbf{AZD4635}}^{\mathbf{A2AR}}$ | nM | A_2A_Ri affinity to A_2A_R | 0.013 | - | Estimated based on *in vitro* data |
| **Tumor dynamics** | | | | | |
| $\mathbf{T}\mathbf{V}_{\mathbf{0}}$ **(CT26, MC38)** | µL | Initial tumor volume | 2.05 | 31.7 | Estimated based on tumor growth data |
| $\boldsymbol{\beta}_{\mathbf{MCA205}}\mathbf{(T}\mathbf{V}_{\mathbf{0}}\mathbf{)}$ | µL | Initial tumor volume | 0.69 | - | Fixed based on experimental conditions |
| **TVmax** | µL | Maximal size of tumor | 3500 | - | Assumed |
| **d0** | d-1 | Spontaneous death rate of tumor cells | 0.01 | - | From (1) |
| **r** | d-1 | Tumor growth rate | 0.522 | 10 | Estimated based on tumor growth data (32) |
| **T cell dynamics** | | | | | |
| **k_pro_** | d^-1^ | ^n^T_eff_ proliferation rate constant | 3.0 | - | From (1) |
| **k_dif_** | d^-1^ | ^n^T_eff_ differentiation rate constant | 3.2 | - | From (1) |
| **k_el_** | d^-1^ | ^n^T_eff_ elimination rate constant | 0.2 | - | From (1) |
| **k_apo_** | d^-1^ | ^d^T_eff_ apoptosis rate constant | 2.0 | - | From (1) |
| **bef** | d^-1^ **•** cell^-1^ | Rate of tumor cell kill by ^d^T_eff_ | 0.001 | - | From (1) |
| **S_L_ (CIV151)** | µL/d | T cell ability to infiltrate tumor tissue under systemic antigen exposure | 4.56 | 24.3 | Estimated based on tumor growth data (32) |
| $\boldsymbol{\beta}_{\mathbf{CIV226}}\mathbf{(}\mathbf{S}_{\mathbf{L}}\mathbf{)}$ | - |  | -0.737 | 15.8 |  |
| $\boldsymbol{\beta}_{\mathbf{CIV227}}\mathbf{(}\mathbf{S}_{\mathbf{L}}\mathbf{)}$ |  |  | -0.747 | 15.2 |  |
| $\boldsymbol{\beta}_{\mathbf{CIV258}}\mathbf{(}\mathbf{S}_{\mathbf{L}}\mathbf{)}$ |  |  | 0.261 | 31.5 |  |
| **Ω_sL_** | - | Random effects on$\mathbf{S}_{\mathbf{L}}$ | 0.322 | 7.47 | Estimated based on tumor growth data (32) |
| **k_LN_** | cells/d | Maximal influx rate of *^n^T_eff_* cells | 210 | 5.47 | Estimated based on tumor growth data (32) |
| $\mathbf{A}\mathbf{g}_{\mathbf{norm}}$ | - | Vaccine impact on Ag_sys_ supply | 1 | - | Control parameter, fixed |
| TME | | | | | |
| **k_pdl_** | d^-1^ | PD-L1 up-regulation rate constant | 1.0 |  | From (1) |
| **K_p_** | cells | Sensitivity of PD-L1 expression up-regulation to ^d^T_eff_ count | 478 | - | From (1) |
| **k_ado_** | d^-1^ | Adenosine accumulation rate constant | 1 | - | Assumed |
| $\mathbf{Vma}\mathbf{x}_{\mathbf{Ado}}$ **(CT26)** | µM | Average adenosine level in tumor | 100 | - | Fixed based on experimental data (19) |
| $\boldsymbol{\beta}_{\mathbf{MC38}}$  $\mathbf{(Vma}\mathbf{x}_{\mathbf{Ado}}\mathbf{)}$ | - |  | -0.5 | - |  |
| $\boldsymbol{\beta}_{\mathbf{MCA205}}$  $\mathbf{(Vma}\mathbf{x}_{\mathbf{Ado}}\mathbf{)}$ | - |  | -3 | - |  |
| $\mathbf{sc}\mathbf{F}_{\mathbf{Ado}}$ | n/a | Scaling factor for adenosine calculation in effect compartment | 1 | - | Selected based on tumor growth data (32), Table 1 |
| $\mathbf{Fmax}_{\mathbf{A2Rs}}$ |  | Assumption of total inhibition of immune cell functions under high adenosine concentrations (36) | 1 | - | Set based on *in vitro* data (36) |
| $\mathbf{Kd}_{\mathbf{Ado}}^{\mathbf{A2AR}}$ | µM | Adenosine affinity to A_2A_R | 1.182 | - | Estimated based on *in vitro* data (19) |
| **S_R_ (CT26)** | µL/d | Sensitivity of cellular immunosuppression to build-up of systemic Ag | 57.1 | 38.5 | Estimated based on tumor growth data (32) |
| $\boldsymbol{\beta}_{\mathbf{MC38}}\mathbf{(}\mathbf{S}_{\mathbf{R}}\mathbf{)}$ | - |  | -0.156 | 53.8 |  |
| $\boldsymbol{\beta}_{\mathbf{MCA205}}\mathbf{(}\mathbf{S}_{\mathbf{LR}}\mathbf{)}$ |  |  | 0.531 | 15.6 |  |
| **Ω_sR_** | - | Random effects on$\mathbf{S}_{\mathbf{R}}$ | 0.137 | 29.4 |  |
| **b** | n/a | Proportional component of residual error | 0.271 | 3.02 | Estimated based on tumor growth data (32) |

## Model diagnostics

Pre-specified diagnostic plots were used to evaluate the quality of data reproduction by the full model. Observations *vs.* model predictions as well as distributions and scatterplots of the residuals are summarized in Figure S2.


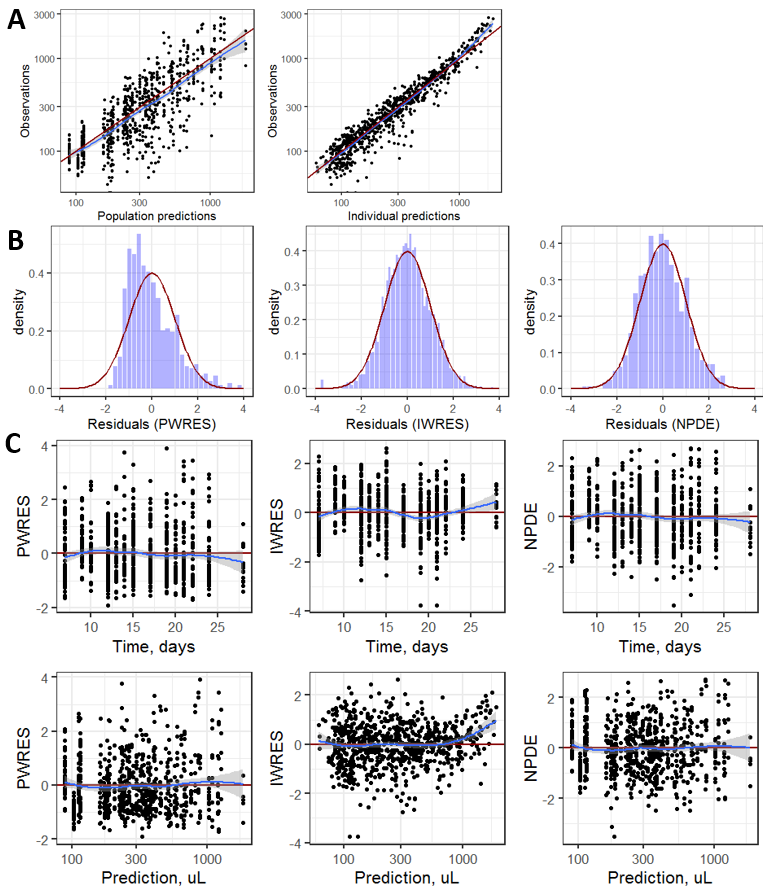


***Figure S2. Model diagnostic plots.*** *(****A)*** *Observations vs. population- and individual-level model predictions;* ***(B)*** *Residuals distribution;* ***(C)*** *Scatterplots of the residuals. Red lines denote an ideal match between experimental data and model predictions; blue lines indicate empirical data trend.*

The model provided unbiased reproduction of the entire dataset (Figure S1). As seen in Figure S1A, use of individually estimated parameters sL and sR allowed for a more accurate reproduction of the experimental data. This conclusion is also supported by a comparison of experimental data time courses *vs.* population- and individual-level model simulations (Figure S3).


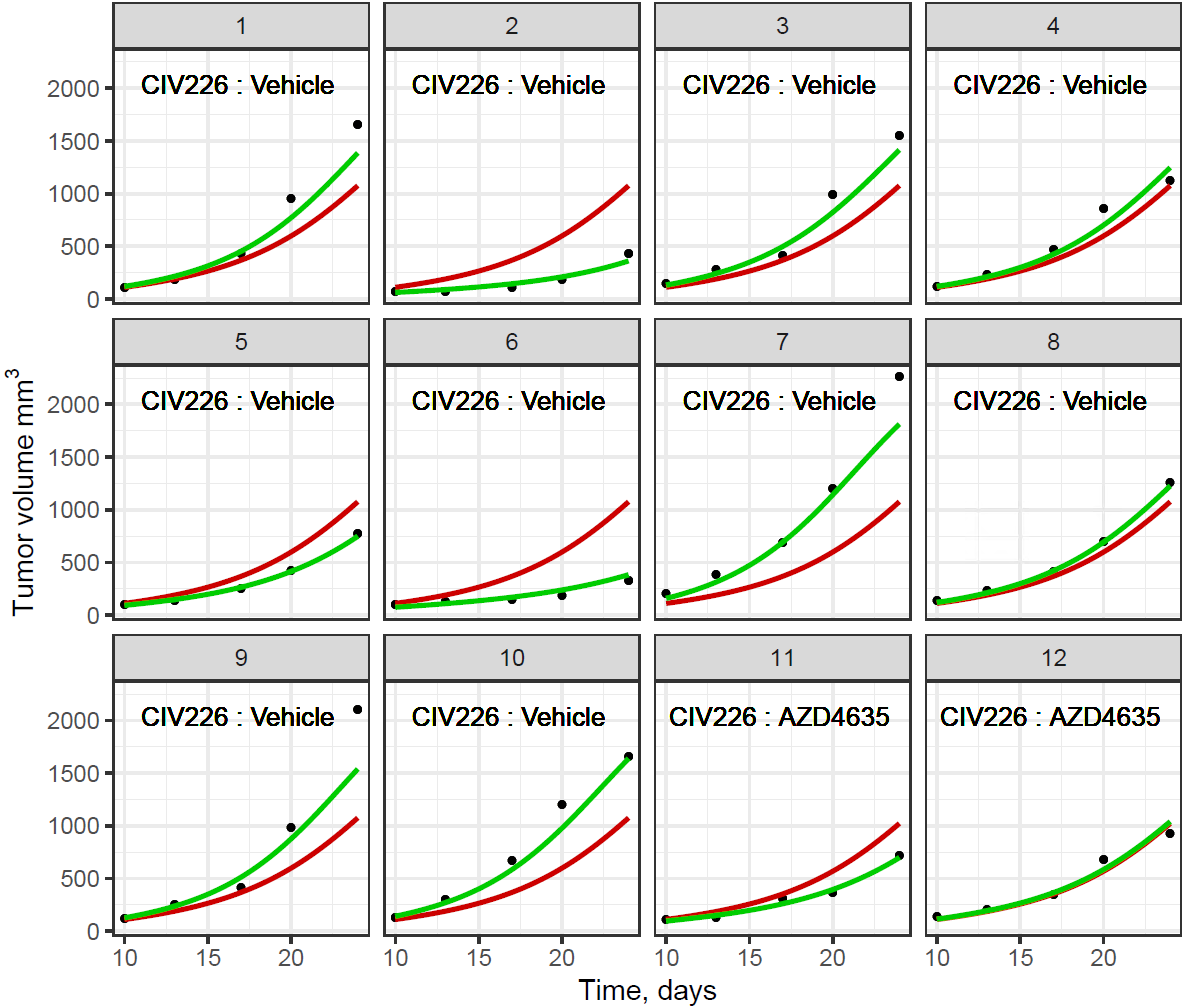

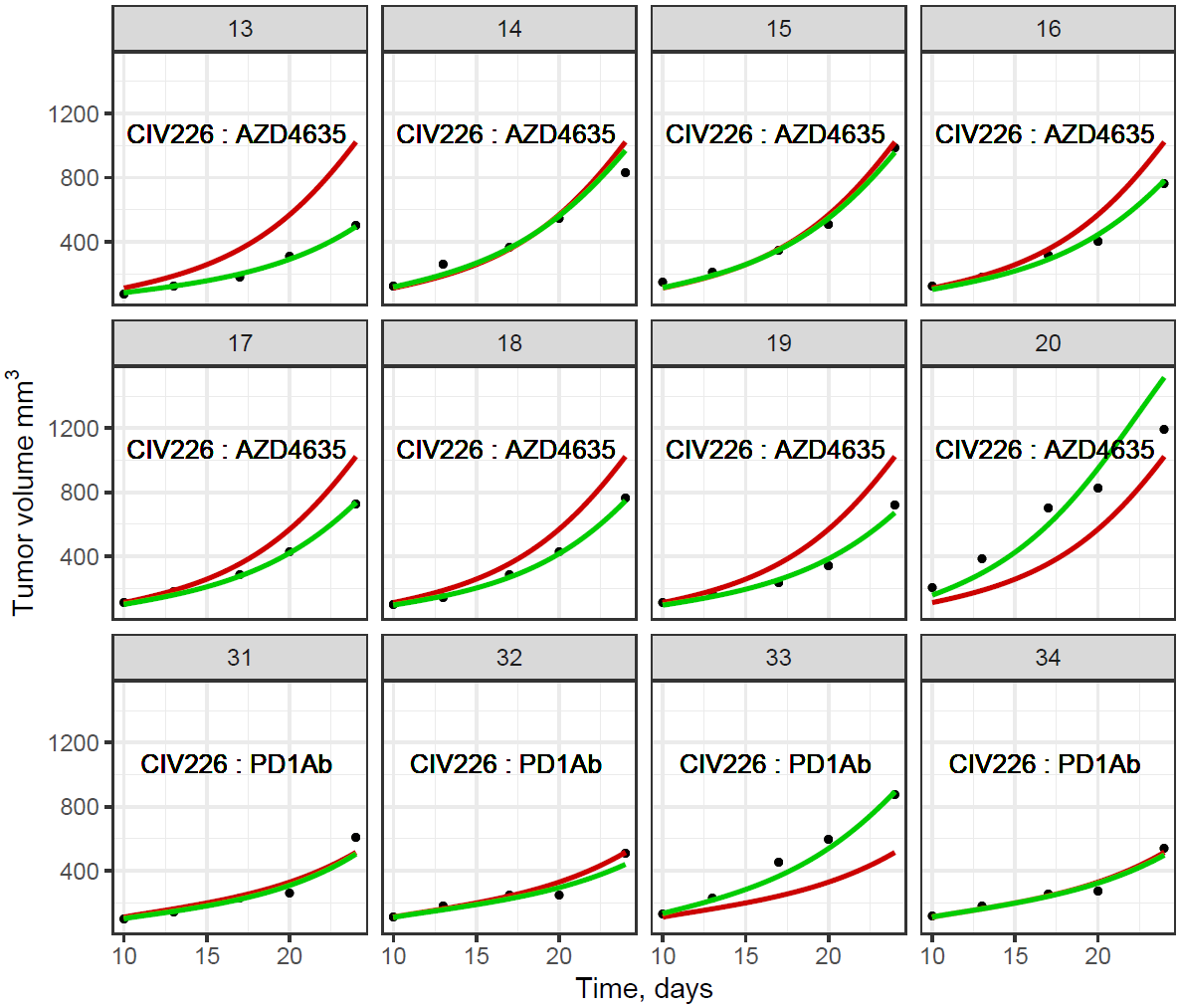


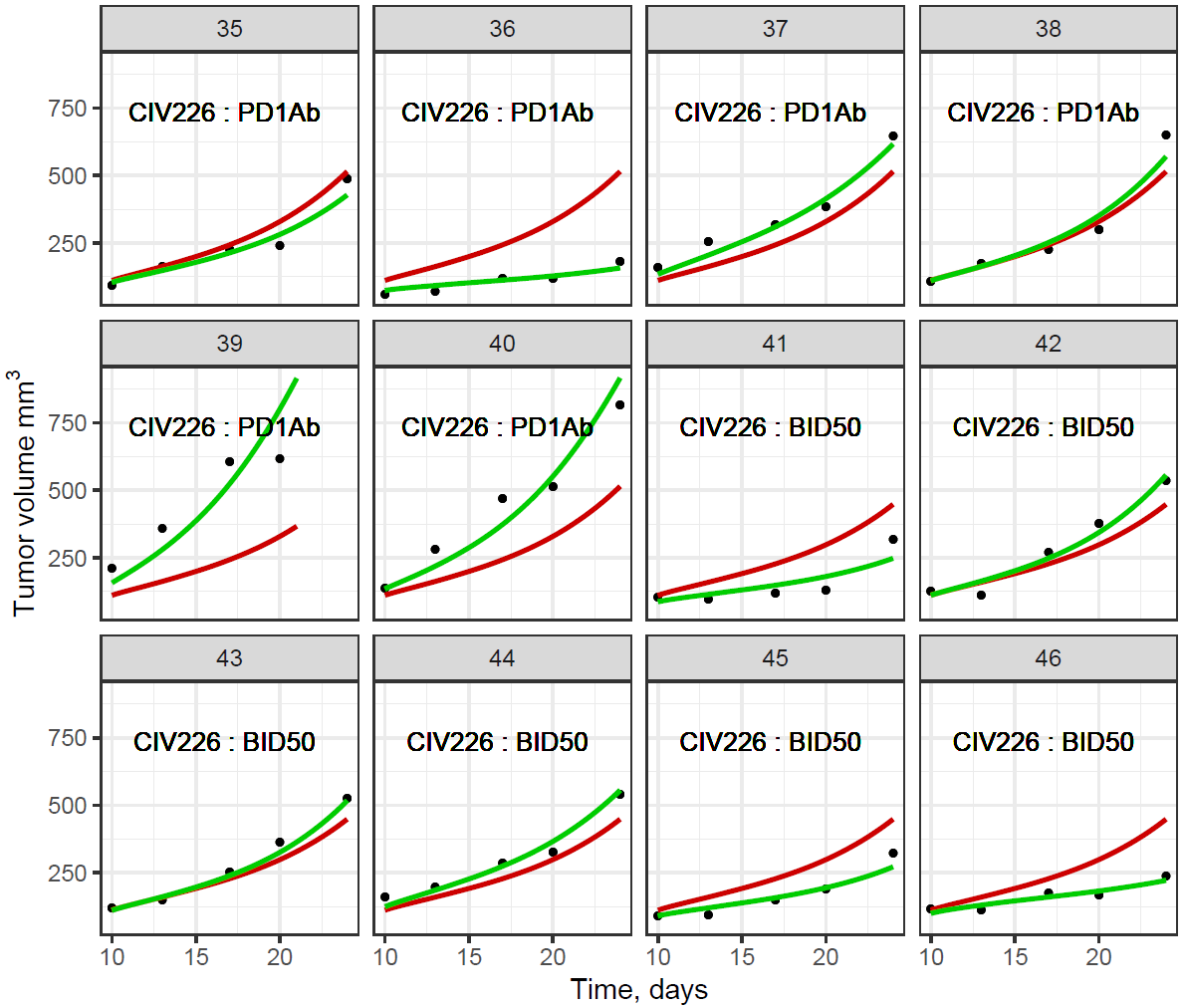

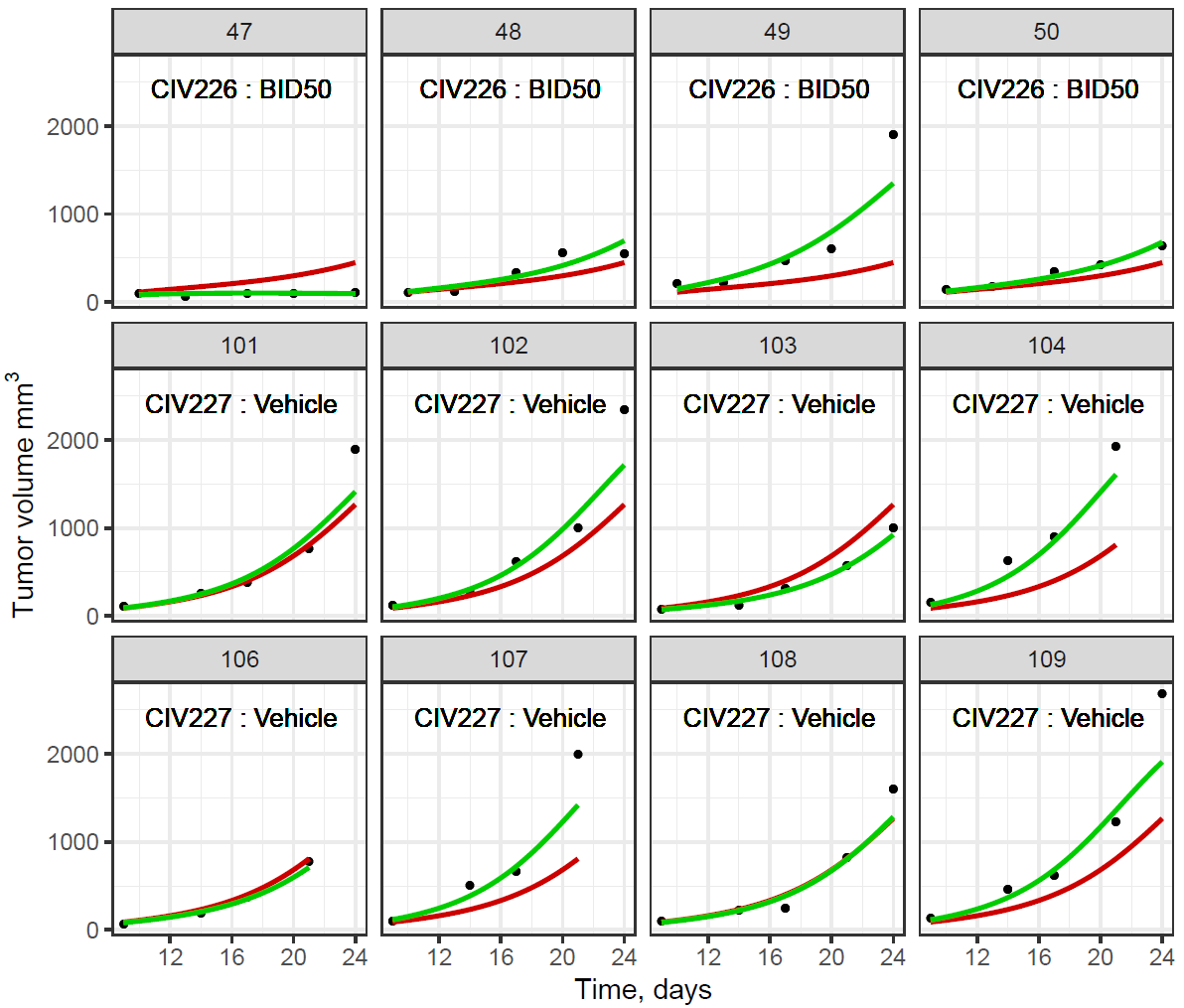


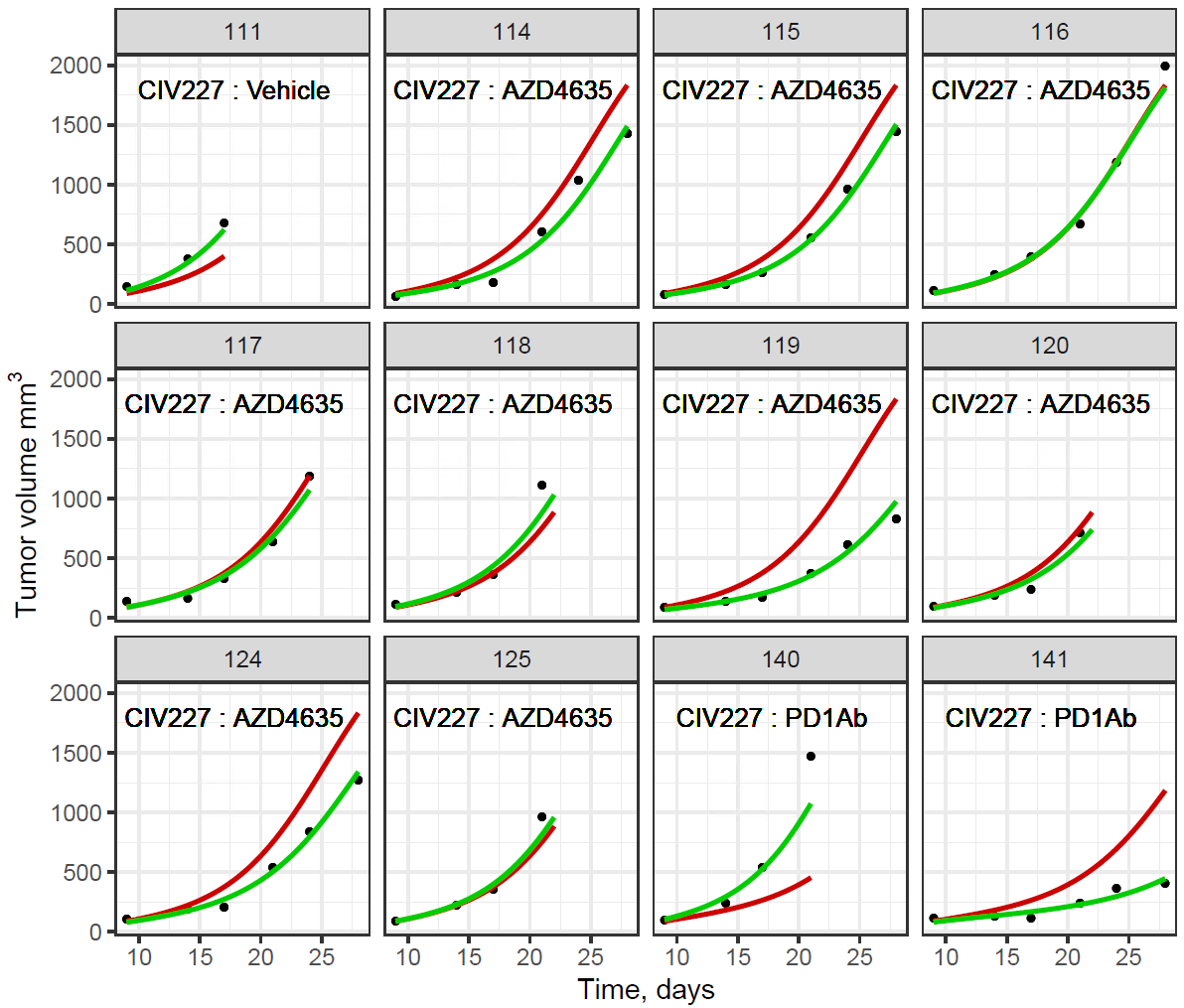

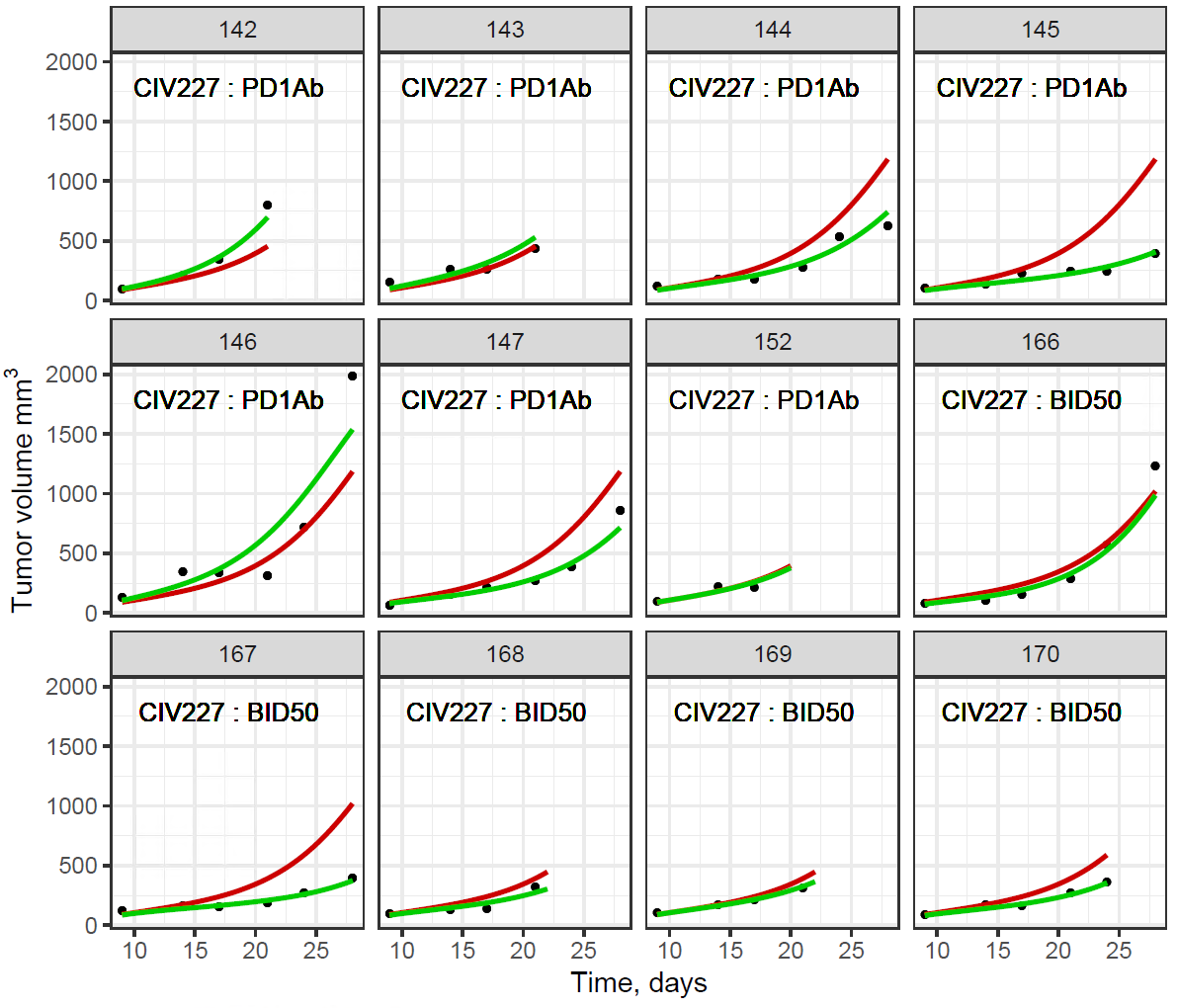


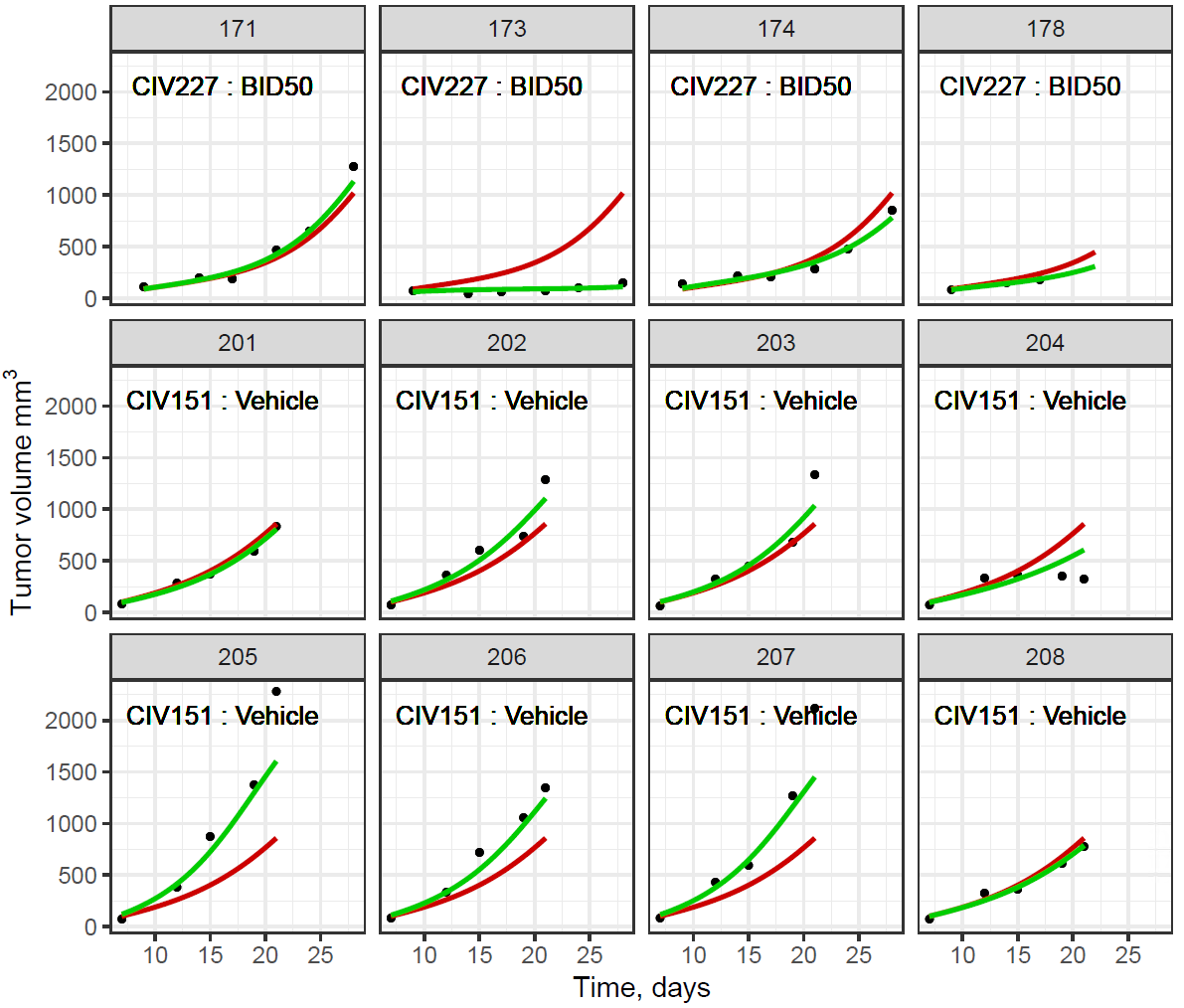

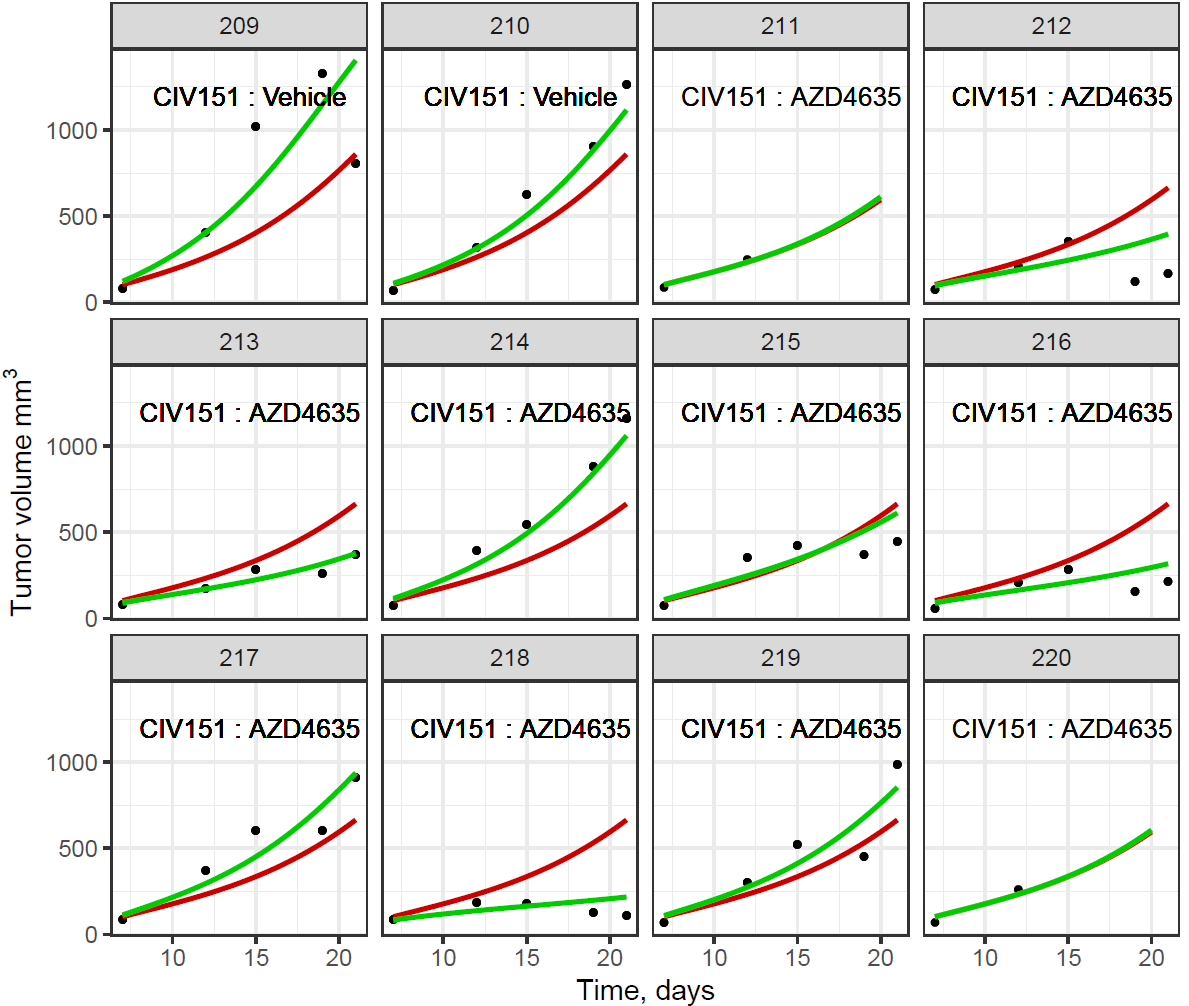

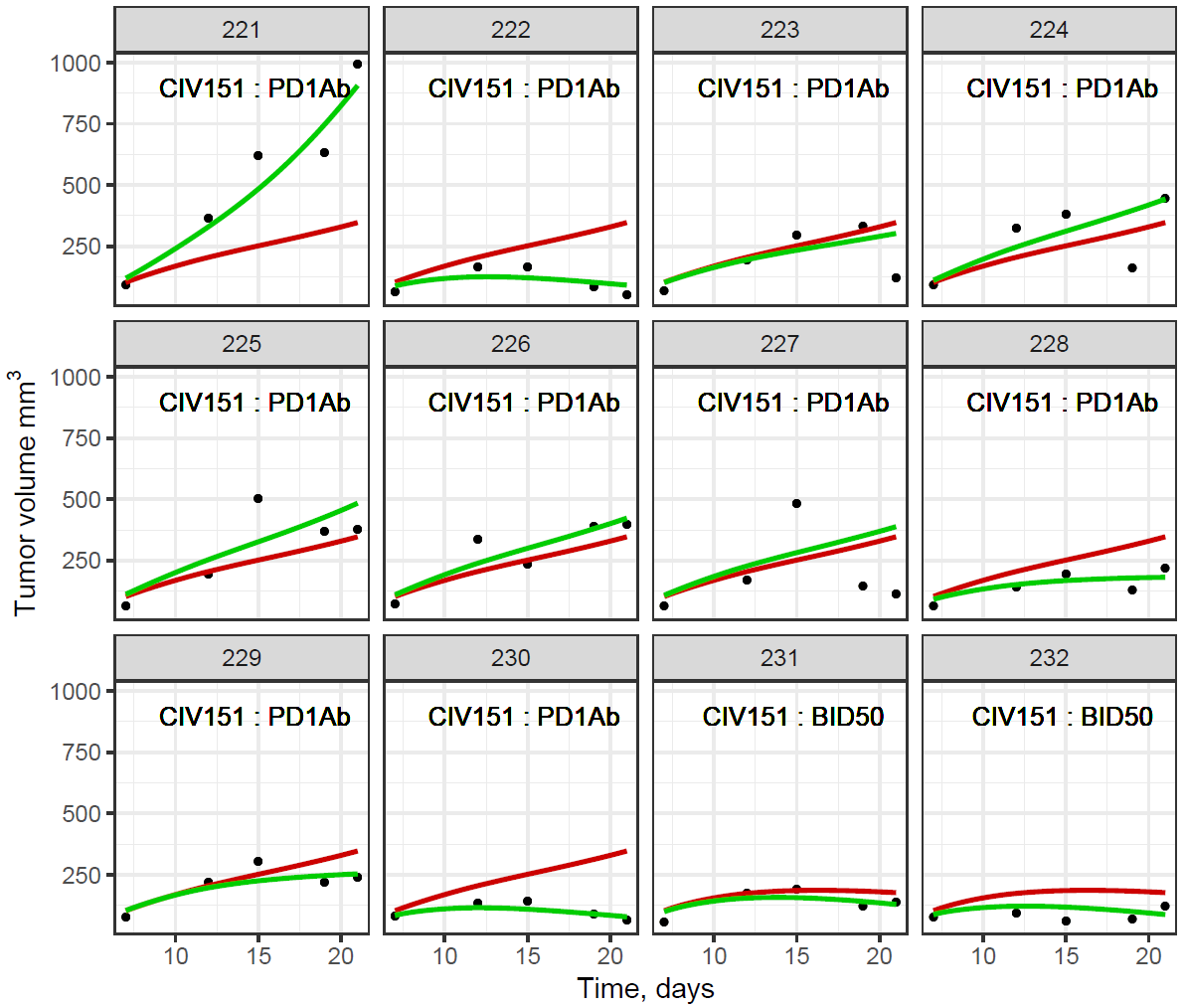

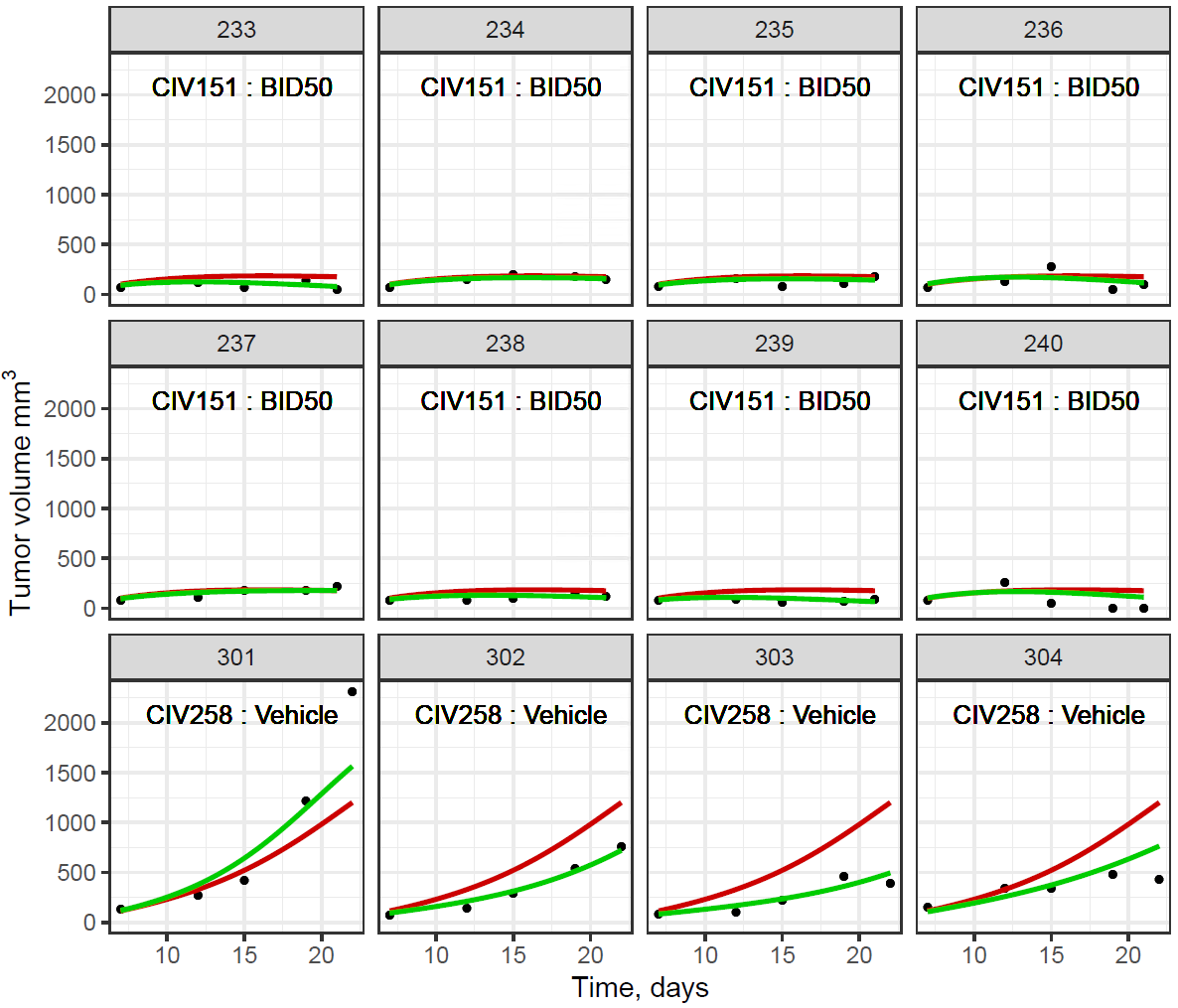


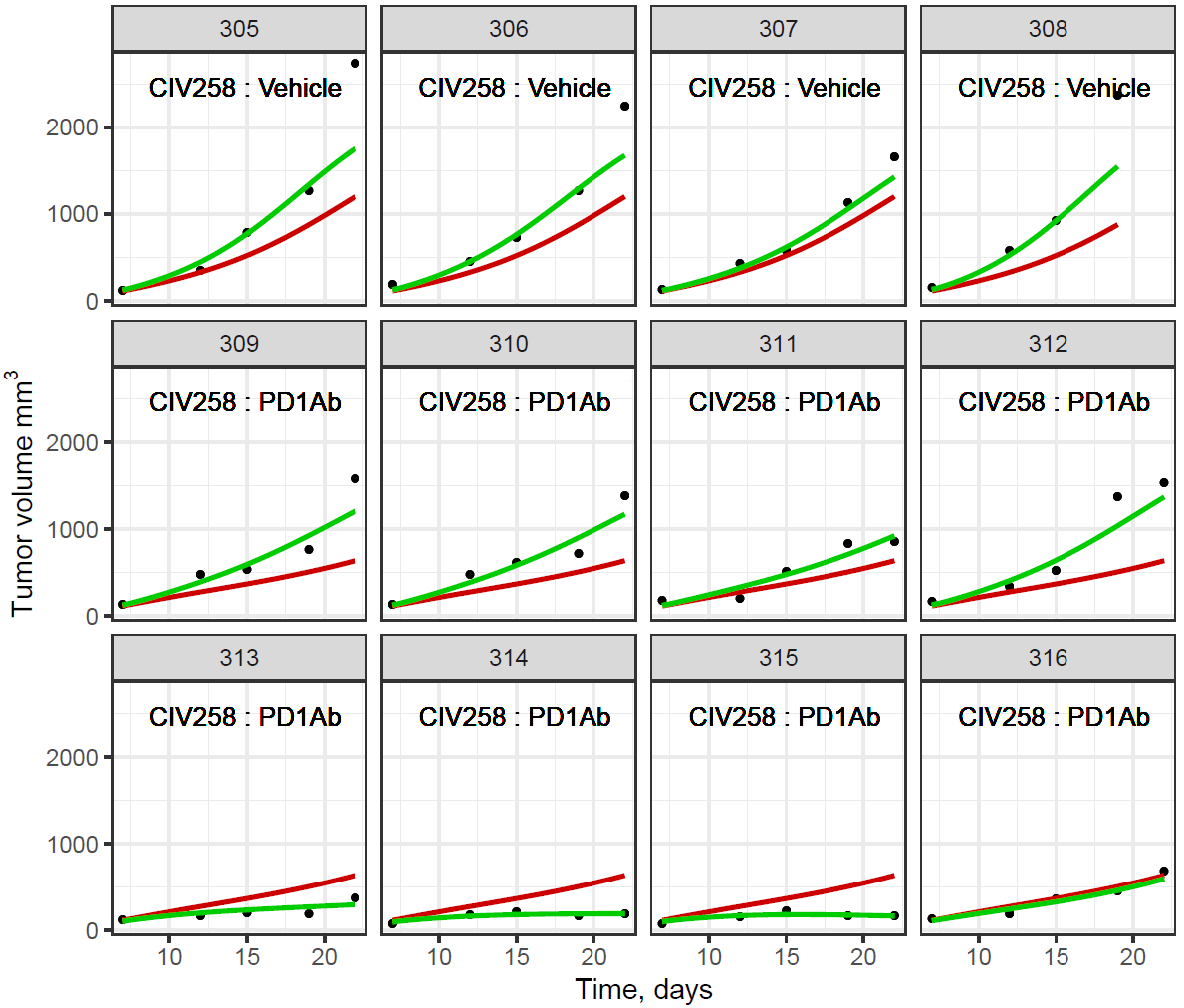

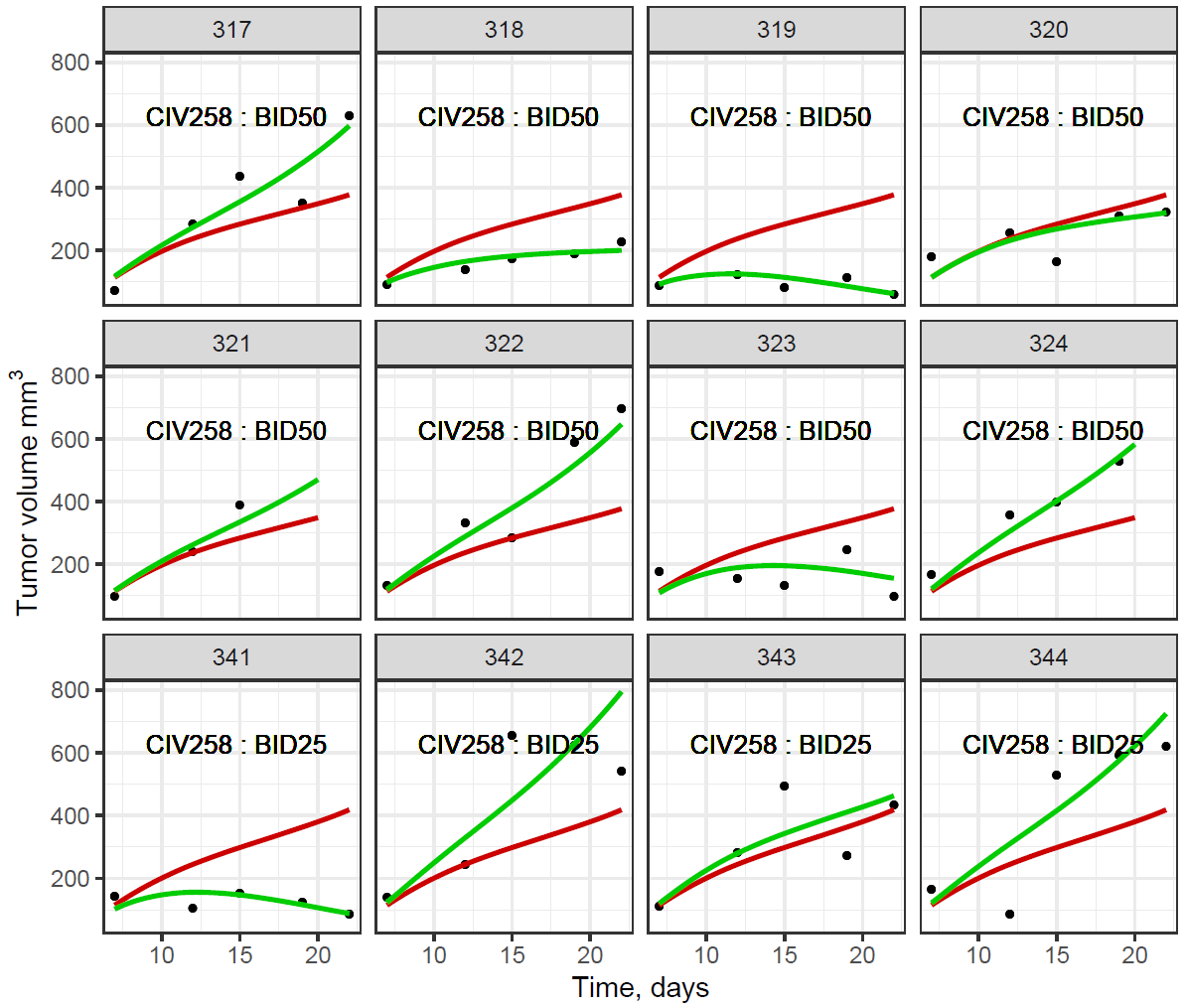


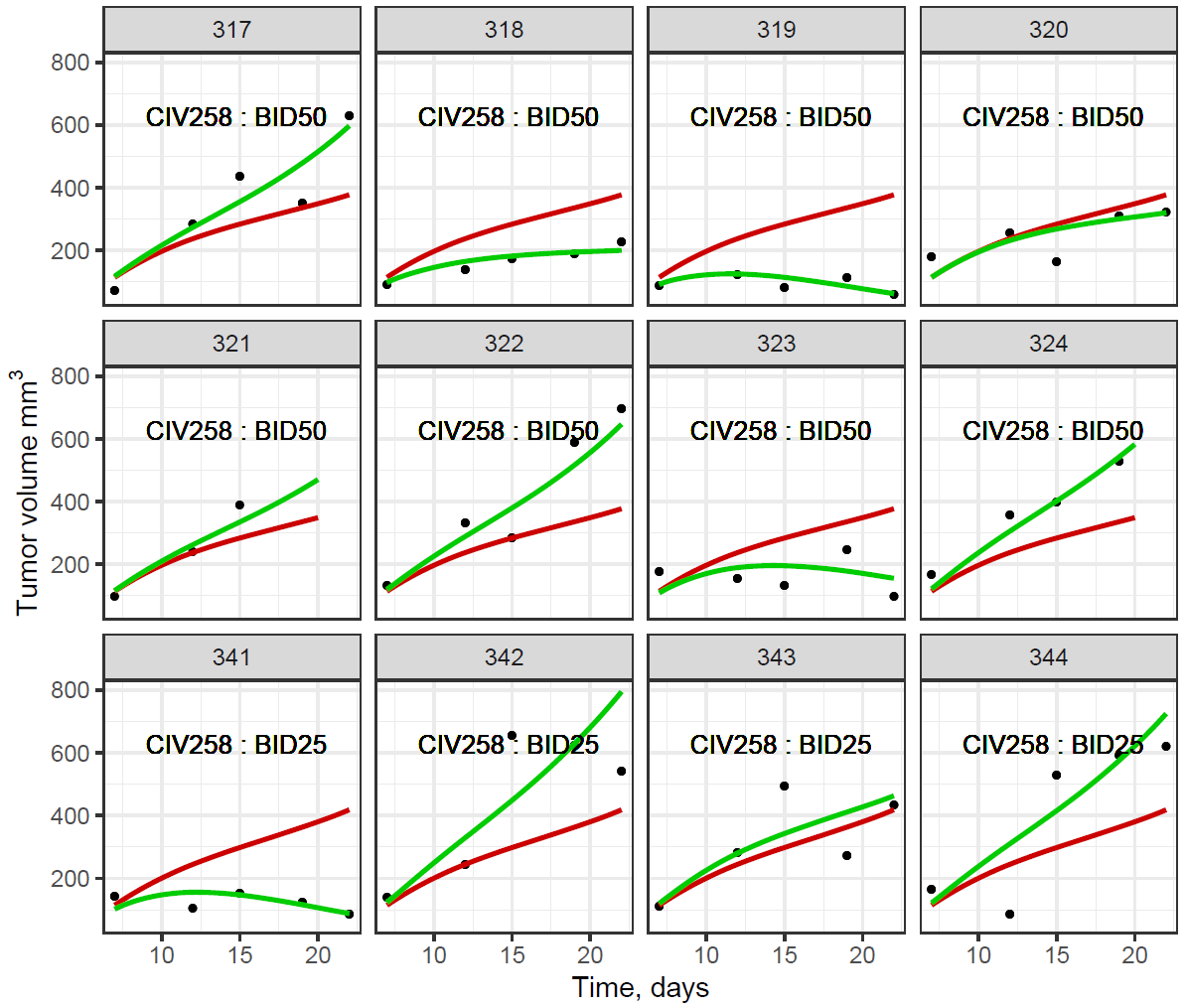


***Figure S3.*** ***Evaluation of model predictions against individual experimental data.*** *Black dots: tumor size dynamics data; red lines: model predictions, population-level; green lines: model predictions, individual-level.*

## PK and *in vitro* data reproduction

Pharmacokinetic and binding properties of AZD4635 were adequately captured by the proposed model (Figure S4):


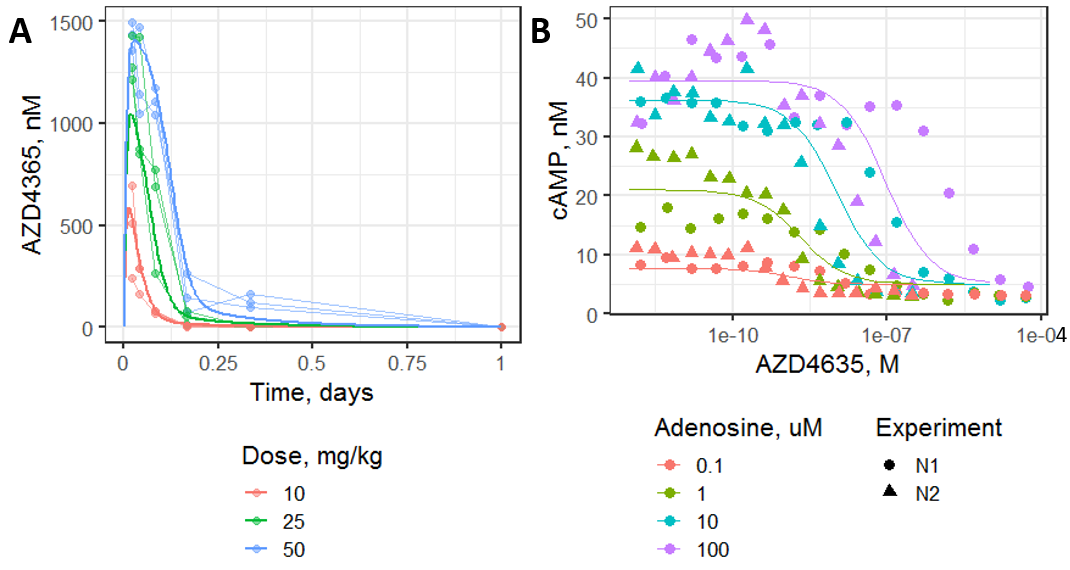


***Figure S4. Experimental data reproduction by the model.*** ***(A)*** *AZD4635 concentration in blood plasma of BALBc mice after a single administration of different AZD4635 doses (marked by colors);* ***(B)*** *cAMP production during cell exposure to different AZD4635 and adenosine concentrations (marked by colors); symbols denote data from individual experiments; lines indicate model simulations.*

# Additional figures


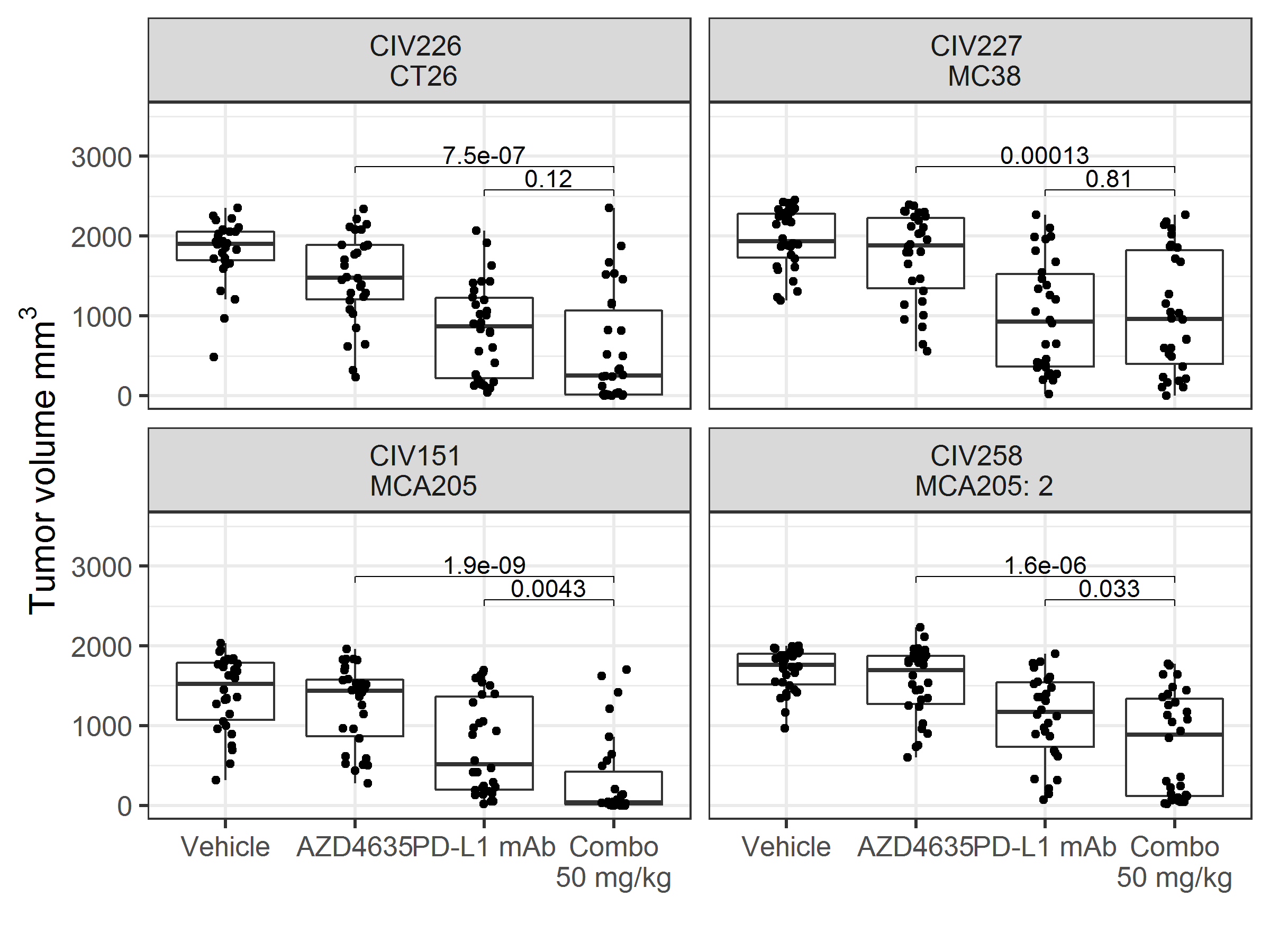


***Figure S5.*** *Predicted tumor volume at the end of the treatment. A tatistical comparison was performed using Student’s t-test; 30 animals per treatment group were used for the simulations*


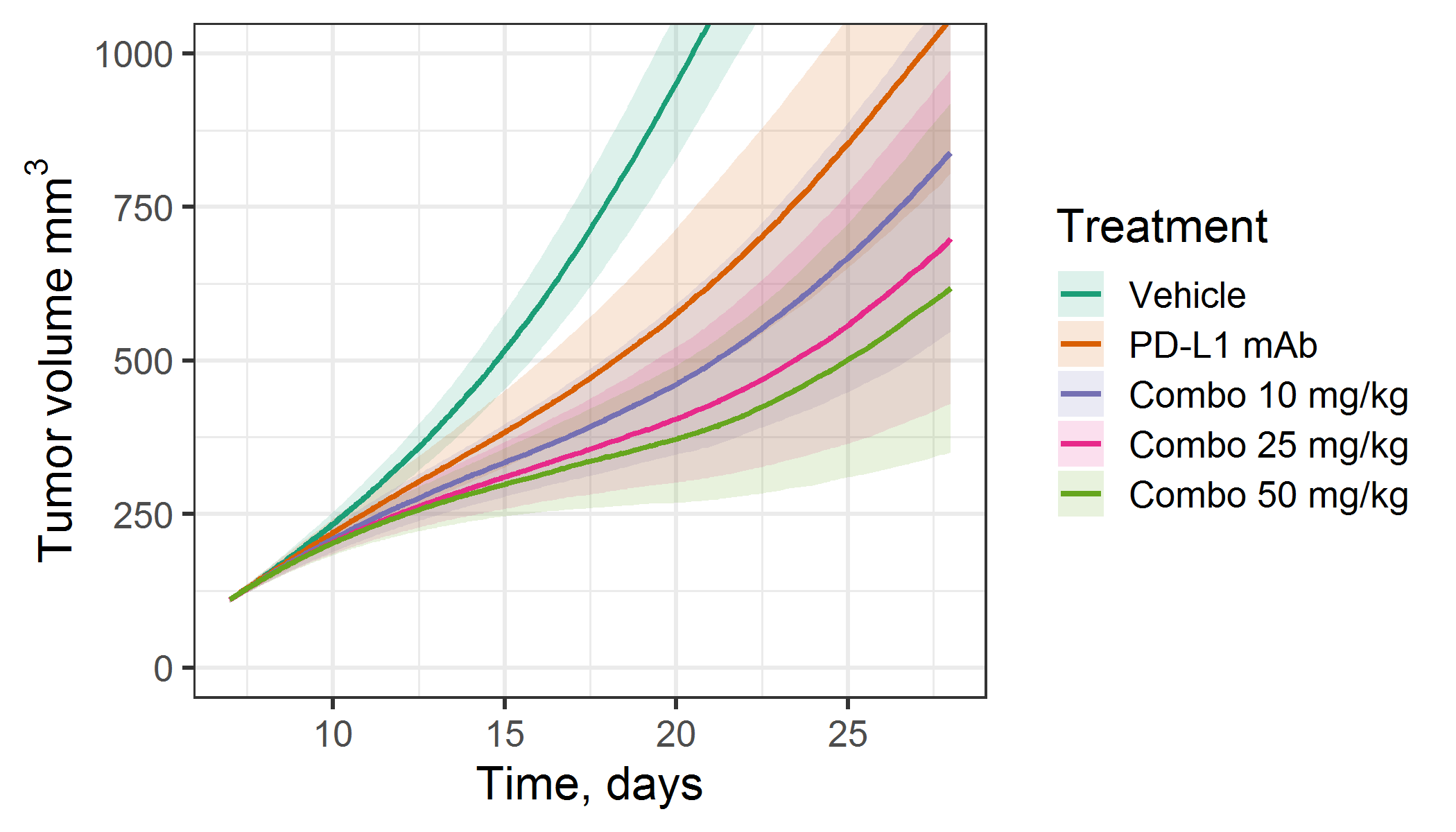


***Figure S6.*** *Distributions of model predictions (population-level) for a dose-finding study in a MCA205 syngeneic model (median prediction and 30% confidence intervals)*


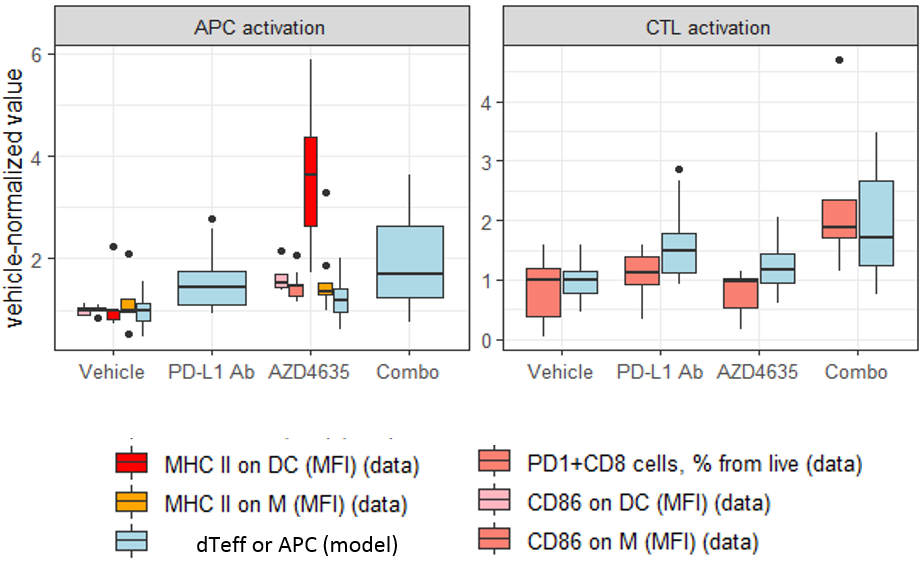


***Figure S7.*** *Model validation using flow cytometry data from* (32)*. A biopsy was taken on Day 14, post-treatment start; various markers were used to evaluate APC or Teff functions in the experimental settings. Model-based simulations are shown in blue color; experimental data are in red/orange color.*


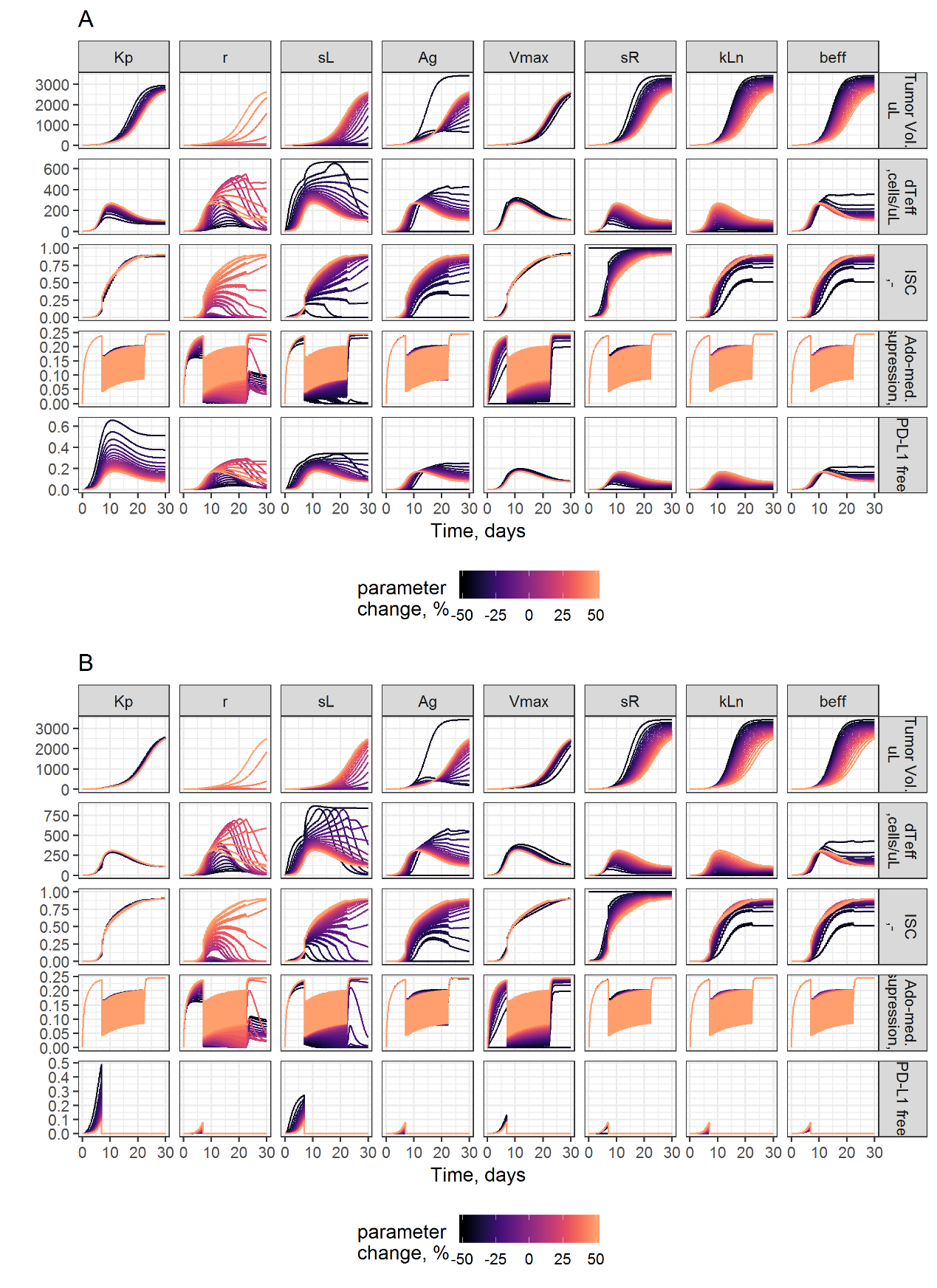


***Figure S8. Model sensitivity analysis.*** *Parameter values were varied, one by one, in a pre-defined range (±50%), and the time* *dynamics of TME components under* ***(A****) AZD4635 alone or* ***(B)*** *in combination with ant-PD-L1 mAb were simulated. Colors denote simulations obtained using different parameter values.*

# Reference list

1. Kosinsky Y, Dovedi SJ, Peskov K, Voronova V, Chu L, Tomkinson H, Al-Huniti N, Stanski DR, Helmlinger G. Radiation and PD-(L)1 treatment combinations: immune response and dose optimization via a predictive systems model. *J Immunother Cancer* (2018) **6**: doi:10.1186/s40425-018-0327-9

2. Law AMK, Lim E, Ormandy CJ, Gallego-Ortega D. The innate and adaptive infiltrating immune systems as targets for breast cancer immunotherapy. *Endocr Relat Cancer* (2017) **24**:R123–R144. doi:10.1530/ERC-16-0404

3. Stagg J, Beavis PA, Divisekera U, Liu MCP, Moller A, Darcy PK, Smyth MJ. CD73-Deficient Mice Are Resistant to Carcinogenesis. *Cancer Res* (2012) **72**:2190–2196. doi:10.1158/0008-5472.CAN-12-0420

4. Mittal D, Young A, Stannard K, Yong M, Teng MWL, Allard B, Stagg J, Smyth MJ. Antimetastatic Effects of Blocking PD-1 and the Adenosine A2A Receptor. *Cancer Res* (2014) **74**:3652–3658. doi:10.1158/0008-5472.CAN-14-0957

5. Chen DS, Mellman I. Oncology Meets Immunology: The Cancer-Immunity Cycle. *Immunity* (2013) **39**:1–10. doi:10.1016/j.immuni.2013.07.012

6. Lechner MG, Karimi SS, Barry-Holson K, Angell TE, Murphy KA, Church CH, Ohlfest JR, Hu P, Epstein AL. Immunogenicity of Murine Solid Tumor Models as a Defining Feature of In Vivo Behavior and Response to Immunotherapy: *J Immunother* (2013) **36**:477–489. doi:10.1097/01.cji.0000436722.46675.4a

7. Mosely SIS, Prime JE, Sainson RCA, Koopmann J-O, Wang DYQ, Greenawalt DM, Ahdesmaki MJ, Leyland R, Mullins S, Pacelli L, et al. Rational Selection of Syngeneic Preclinical Tumor Models for Immunotherapeutic Drug Discovery. *Cancer Immunol Res* (2017) **5**:29–41. doi:10.1158/2326-6066.CIR-16-0114

8. Pardoll DM. The blockade of immune checkpoints in cancer immunotherapy. *Nat Rev Cancer* (2012) **12**:252–264. doi:10.1038/nrc3239

9. Adams JL, Smothers J, Srinivasan R, Hoos A. Big opportunities for small molecules in immuno-oncology. *Nat Rev Drug Discov* (2015) **14**:603–622. doi:10.1038/nrd4596

10. Wang C, Thudium KB, Han M, Wang X-T, Huang H, Feingersh D, Garcia C, Wu Y, Kuhne M, Srinivasan M, et al. In Vitro Characterization of the Anti-PD-1 Antibody Nivolumab, BMS-936558, and In Vivo Toxicology in Non-Human Primates. *Cancer Immunol Res* (2014) **2**:846–856. doi:10.1158/2326-6066.CIR-14-0040

11. Gowrishankar K, Gunatilake D, Gallagher SJ, Tiffen J, Rizos H, Hersey P. Inducible but Not Constitutive Expression of PD-L1 in Human Melanoma Cells Is Dependent on Activation of NF-κB. *PLOS ONE* (2015) **10**:e0123410. doi:10.1371/journal.pone.0123410

12. Leone RD, Lo Y-C, Powell JD. A2aR antagonists: Next generation checkpoint blockade for cancer immunotherapy. *Comput Struct Biotechnol J* (2015) **13**:265–272. doi:10.1016/j.csbj.2015.03.008

13. Fredholm BB. Adenosine, an endogenous distress signal, modulates tissue damage and repair. *Cell Death Differ* (2007) **14**:1315–1323. doi:10.1038/sj.cdd.4402132

14. Loi S, Pommey S, Haibe-Kains B, Beavis PA, Darcy PK, Smyth MJ, Stagg J. CD73 promotes anthracycline resistance and poor prognosis in triple negative breast cancer. *Proc Natl Acad Sci* (2013) **110**:11091–11096. doi:10.1073/pnas.1222251110

15. Morote-Garcia JC, Rosenberger P, Kuhlicke J, Eltzschig HK. HIF-1-dependent repression of adenosine kinase attenuates hypoxia-induced vascular leak. *Blood* (2008) **111**:5571–5580. doi:10.1182/blood-2007-11-126763

16. Hatfield SM, Kjaergaard J, Lukashev D, Belikoff B, Schreiber TH, Sethumadhavan S, Abbott R, Philbrook P, Thayer M, Shujia D, et al. Systemic oxygenation weakens the hypoxia and hypoxia inducible factor 1α-dependent and extracellular adenosine-mediated tumor protection. *J Mol Med* (2014) **92**:1283–1292. doi:10.1007/s00109-014-1189-3

17. Hatfield SM, Sitkovsky M. A2A adenosine receptor antagonists to weaken the hypoxia-HIF-1α driven immunosuppression and improve immunotherapies of cancer. *Curr Opin Pharmacol* (2016) **29**:90–96. doi:10.1016/j.coph.2016.06.009

18. Blay J, White TD, Hoskin DW. The extracellular fluid of solid carcinomas contains immunosuppressive concentrations of adenosine. *Cancer Res* (1997) **57**:2602–2605.

19. Borodovsky A, Wang Y, Ye M, Shaw JC, Sachsenmeier K, Deng N, Goodwin K, Clarke JD, Goodwin R, Strittmatter N, et al. Abstract 3751: Inhibition of A2AR by AZD4635 induces anti-tumor immunity alone and in combination with anti-PD-L1 in preclinical models. *Cancer Res* (2018) **78**:3751–3751. doi:10.1158/1538-7445.AM2018-3751

20. Willingham SB, Ho PY, Hotson A, Hill C, Piccione EC, Hsieh J, Liu L, Buggy JJ, McCaffery I, Miller RA. A2AR Antagonism with CPI-444 Induces Antitumor Responses and Augments Efficacy to Anti–PD-(L)1 and Anti–CTLA-4 in Preclinical Models. *Cancer Immunol Res* (2018) **6**:1136–1149. doi:10.1158/2326-6066.CIR-18-0056

21. Allard D, Turcotte M, Stagg J. Targeting A2 adenosine receptors in cancer. *Immunol Cell Biol* (2017) **95**:333–339. doi:10.1038/icb.2017.8

22. Murphree LJ, Sullivan GW, Marshall MA, Linden J. Lipopolysaccharide rapidly modifies adenosine receptor transcripts in murine and human macrophages: role of NF-κB in A _2A_ adenosine receptor induction. *Biochem J* (2005) **391**:575–580. doi:10.1042/BJ20050888

23. Kjaergaard J, Hatfield S, Jones G, Ohta A, Sitkovsky M. A2A Adenosine Receptor Gene Deletion or Synthetic A2A Antagonist Liberate Tumor-Reactive CD8 + T Cells from Tumor-Induced Immunosuppression. *J Immunol* (2018)ji1700850. doi:10.4049/jimmunol.1700850

24. Cekic C, Sag D, Li Y, Theodorescu D, Strieter RM, Linden J. Adenosine A2B Receptor Blockade Slows Growth of Bladder and Breast Tumors. *J Immunol* (2012) **188**:198–205. doi:10.4049/jimmunol.1101845

25. Iannone R, Miele L, Maiolino P, Pinto A, Morello S. Blockade of A2b adenosine receptor reduces tumor growth and immune suppression mediated by myeloid-derived suppressor cells in a mouse model of melanoma. *Neoplasia N Y N* (2013) **15**:1400–1409.

26. Wilson JM, Ross WG, Agbai ON, Frazier R, Figler RA, Rieger J, Linden J, Ernst PB. The A2B Adenosine Receptor Impairs the Maturation and Immunogenicity of Dendritic Cells. *J Immunol* (2009) **182**:4616–4623. doi:10.4049/jimmunol.0801279

27. Novitskiy SV, Ryzhov S, Zaynagetdinov R, Goldstein AE, Huang Y, Tikhomirov OY, Blackburn MR, Biaggioni I, Carbone DP, Feoktistov I, et al. Adenosine receptors in regulation of dendritic cell differentiation and function. *Blood* (2008) **112**:1822–1831. doi:10.1182/blood-2008-02-136325

28. Alicea-Torres K, Gabrilovich DI. “Biology of Myeloid-Derived Suppressor Cells,” in *Oncoimmunology*, eds. L. Zitvogel, G. Kroemer (Cham: Springer International Publishing), 181–197. Available at: http://link.springer.com/10.1007/978-3-319-62431-0_10 [Accessed June 20, 2018]

29. Hoskin DW, Mader JS, Furlong SJ, Conrad DM, Blay J. Inhibition of T cell and natural killer cell function by adenosine and its contribution to immune evasion by tumor cells (Review). *Int J Oncol* (2008) **32**:527–535.

30. Hovi T, Smyth JF, Allison AC, Williams SC. Role of adenosine deaminase in lymphocyte proliferation. *Clin Exp Immunol* (1976) **23**:395–403.

31. Yu JW, Bhattacharya S, Yanamandra N, Kilian D, Shi H, Yadavilli S, Katlinskaya Y, Kaczynski H, Conner M, Benson W, et al. Tumor-immune profiling of murine syngeneic tumor models as a framework to guide mechanistic studies and predict therapy response in distinct tumor microenvironments. *PLOS ONE* (2018) **13**:e0206223. doi:10.1371/journal.pone.0206223

32. Borodovsky A, Barbon CM, Wang Y, Ye M, Prickett L, Chandra D, Shaw J, Deng N, Sachsenmeier K, Clarke JD, et al. Small molecule AZD4635 inhibitor of A 2AR signaling rescues immune cell function including CD103+ dendritic cells enhancing anti-tumor immunity. *J Immunother Cancer* (2020) **8**:e000417. doi:10.1136/jitc-2019-000417

33. Salahudeen MS, Nishtala PS. An overview of pharmacodynamic modelling, ligand-binding approach and its application in clinical practice. *Saudi Pharm J* (2017) **25**:165–175. doi:10.1016/j.jsps.2016.07.002

34. Bonate PL, Desai A, Rizwan A, Lu Z, Tannenbaum S. “Nonlinear Mixed Effects Modeling in Systems Pharmacology,” in *Systems Pharmacology and Pharmacodynamics*, eds. D. E. Mager, H. H. C. Kimko (Cham: Springer International Publishing), 255–276. Available at: http://link.springer.com/10.1007/978-3-319-44534-2_12 [Accessed December 10, 2020]

35. National Center for Biotechnology Information. PubChem Compound Summary for CID 86676119. https://pubchem.ncbi.nlm.nih.gov/compound/azd4635. Accessed Aug. 11, 2020.

36. Huang S, Apasov S, Koshiba M, Sitkovsky M. Role of A2a extracellular adenosine receptor-mediated signaling in adenosine-mediated inhibition of T-cell activation and expansion. *Blood* (1997) **90**:1600–1610.
